# Risk factors of neonatal mortality and child mortality in Bangladesh

**DOI:** 10.7189/jogh.08.010421

**Published:** 2018-06

**Authors:** Md Maniruzzaman, Harman S Suri, Nishith Kumar, Md Menhazul Abedin, Md Jahanur Rahman, Ayman El-Baz, Makrand Bhoot, Jagjit S Teji, Jasjit S Suri

**Affiliations:** 1 Department of Statistics, University of Rajshahi, Rajshahi, Bangladesh; 2 The JiVitA Project of John Hopkins University, Gaibandha, Bangladesh; 3 Brown University, Providence, Rhode Island, USA; 4 AtheroPoint LLC, Roseville, California, USA; 5 Department of Statistics, Bangabandhu Sheikh Mujibur Rahman Science and Technology University, Gopalganj, Bangladesh; 6 Statistics Discipline, Khulna University, Khulna, Bangladesh; 7 Department of Bioengineering, University of Louisville, Louisville, Kentucky, USA; 8 Director, Professional Alliance for Technology & Habitat, New York, New York, USA; 9 Neonatologist, Ann and Robert H. Lurie Children’s Hospital of Chicago, Chicago, USA; 10 Epidemiology Department, Global Biomedical Technologies, Inc., Roseville, California, USA; 11 Department of Electrical Engineering, Idaho State University (Affl.), Idaho, USA

## Abstract

**Background:**

Child and neonatal mortality is a serious problem in Bangladesh. The main objective of this study was to determine the most significant socio-economic factors (covariates) between the years 2011 and 2014 that influences on neonatal and child mortality and to further suggest the plausible policy proposals.

**Methods:**

We modeled the neonatal and child mortality as categorical dependent variable (alive vs death of the child) while 16 covariates are used as independent variables using χ^2^ statistic and multiple logistic regression (MLR) based on maximum likelihood estimate.

**Findings:**

Using the MLR, for neonatal mortality, diarrhea showed the highest positive coefficient (β = 1.130; *P* < 0.010) leading to most significant covariate for both 2011 and 2014. The corresponding odds ratios were: 0.323 for both the years. The second most significant covariate in 2011 was birth order between 2-6 years (β = 0.744; *P* < 0.001), while father’s education was negative correlation (β = -0.910; *P* < 0.050). In general, 10 covariates in 2011 and 5 covariates in 2014 were significant, so there was an improvement in socio-economic conditions for neonatal mortality. For child mortality, birth order between 2-6 years and 7 and above years showed the highest positive coefficients (β = 1.042; *P* < 0.010) and (β = 1.285; *P* < 0.050) for 2011. The corresponding odds ratios were: 2.835 and 3.614, respectively. Father's education showed the highest coefficient (β = 0.770; *P* < 0.050) indicating the significant covariate for 2014 and the corresponding odds ratio was 2.160. In general, 6 covariates in 2011 and 4 covariates in 2014 were also significant, so there was also an improvement in socio-economic conditions for child mortality. This study allows policy makers to make appropriate decisions to reduce neonatal and child mortality in Bangladesh.

**Conclusions:**

In 2014, mother’s age and father’s education were also still significant covariates for child mortality. This study allows policy makers to make appropriate decisions to reduce neonatal and child mortality in Bangladesh.

Bangladesh is the most densely populated country in the world. There were about 160 million people all across Bangladesh in 2013 [1]. There are eight administrative regions (see **Figure 1**). Neonatal and child mortality is an important indicator of the development of a country like Bangladesh. From 1990 to 2011, the number of child deaths decreased worldwide from 12 million to 6 million [2]. This indicates that 14000 fewer children died each day in 2011 compared to 1990. However, 19000 children aged less than five years still perished every day in 2011. The report of the Bangladesh Demographic and Health Survey (BDHS 2011 report: https://dhsprogram.com/pubs/pdf/fr265/fr265.pdf) showed that the child mortality rate (those less than 5 years of age) in Bangladesh had dropped from 57 deaths per 1000 live births in 1993 to 28 deaths in 2011. Although child mortality had declined, it was still high compared to other countries in the region. Therefore, it is essential to reduce child mortality.

**Figure 1:**
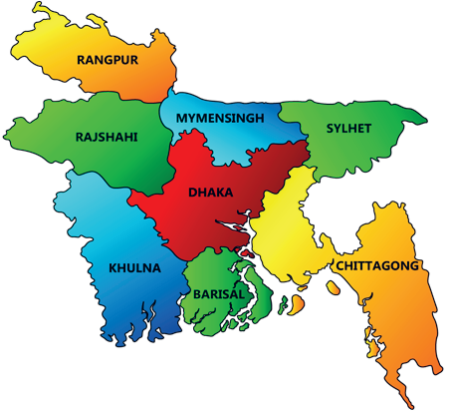
Bangladesh map showing 8 regions.

Muhuri [3], Forste [4], Doctor [5] and Machado et al [6] showed that there was an inverse relationship between socio-economic factors and infant mortality. There was also evidence that mother’s characteristics and a child’s environment were directly related to infant mortality [7]. Parent’s education and occupation was the third pair of factors that have significant influence on neonatal and child mortality in emerging countries [4,8]. Mosley et al [9] showed that the child mortality was directly influenced by mother’s education, type of toilet facility, source of drinking water, maternal and child health care services. Cleland et al [10] studied that early child mortality was highly associated with the mother's education. Breierova et al [11] also showed that both father’s and mother’s education were highly significant for child mortality.

Cleland et al [12] demonstrated that a one year increase in mother's education was associated with 7%-9% reduction in the under-five mortality rate. Gakidou et al [13] found a significant effect of mother’s education on under-five mortality in 175 countries. Similarly, Akter et al [14] showed that there was a positive association between parents’ education and under-five mortality. The factors of infant mortality and child mortality were also studied by Hossain et al [15] and they provided evidence that the determinants were changing according to the age of child. The study also showed that access to health care services, birth interval and mother’s education were the main features of neonatal and child mortality. Abir et al [16] indicated that combined birth rank (less than 2-year interval or greater than 2-year interval) and interval, as well as contraceptive use were the common factors that were associated with child mortality. However, Chowdhury et al [17] studied on the covariates of neonatal and post-neonatal mortality in Bangladesh by using the BDHS 2007 data set and they showed that father’s education, type of place, wealth index, and child’s age were the significant factors for neonatal and child mortality. On the other hand, Hossain et al [18] also conducted studies on child mortality jointly in the Rajshahi and Natore districts.

The main objectives of this paper are: i) To determine the covariates which influence on neonatal and child mortality using χ^2^ and multiple logistic regression (MLR), (ii) To compare of neonatal and child mortality between the years of 2011 and 2014, especially comparing neo11 vs neo14 and child11 against child14, (iii) Understanding the Emerging Risk factors for neonatal and child mortality over time, and (iv) To suggest the plausible policy proposals to reduce the neonatal and child mortality. While these objectives are addressed, the readers will find the following novelties in our study compared to the previous studies:

- The study shows the usage of the latest BDHS 2014 data sets.
- The study will utilize first time 16 covariates compared to previous studies with limited covariance.
- Utilization of the χ^2^-test to show the association between neonatal and child mortality for 16 covariates on the basis of *P*-values.
- Utilization of MLR to identify the risk factors on the basis of odds ratio (OR) and *P*-values.
- Besides the conventional risk factors, we will show new and first time introduced risk factors which are responsible for neonatal and child mortality, demonstrating difference compared to previous studies.
- Comprehensive analysis in understanding the factors which influence on the neonatal and child mortality over time.
- Suggests policy solutions based on the most significant covariates or risk factors to reduce neonatal and child mortality.

The layout of this paper is prepared as follows. Section 1.2 represents Methods. Results are discussed in section 2. Section 3 represents the plausible solutions. Section 4 represents discussion in details and finally a conclusion.

## METHODS

### Input BDHS data

The data sets used in this study were derived from the 2011 and 2014 surveys. The BDHS data set consisted of a total 8753 respondents, taken from 2011 and 7886 respondents, taken from 2014. In 2011, among these, 506 babies died in 0-28 days and 224 babies died under five years of age. In 2014, 240 babies died in 0-28 days and 319 babies died under five years of age. The analysis of this study considered only ever married women of reproductive age (15-49 years) who had at least one live birth before the time of interview. The sampling techniques, survey design, survey instruments, measuring system and quality control have been discussed [19] (BDHS data source: http://www.dhsprogram.com/data/dataset_admin). This study was based on an analysis of existing public domain survey data sets that is freely available online with all identifier information removed. The survey was approved by the Ethics Committee in Bangladesh.

### Tabular representation of the input BDHS data

In the BDHS data set, there were few questions in the questionnaire that were not filled-up by the respondents. As a result, there were some missing values in the data set. In 2011 data set, there were 6 missing values in father’s education levels, 1 missing value in father’s occupation, 7774 in mother’s occupation, and 7395 in succeeding birth interval (SBI), 412 in diarrhea and 11 in mother’s age. On the other hand, in 2014 data set, there were two missing values in father’s education levels, 27 missing values in father’s occupation, 5780 in mother’s education, 6904 in succeeding birth interval (SBI), 1 in type of toilet facility and 326 in diarrhea. Most of the papers did not considered the missing values. However, in this study, we have replaced missing values with the mode for categorical variables and arithmetic mean for numerical variables.

We created **Table 1** for representing the socio-economic characteristics for neonatal and child mortality. The first column represents the attributes of the selected covariates. The second and fourth columns represent the number of neonatal death (alive vs death) and the third and fifth columns represent child death (alive vs death). We will use the following abbreviations: neo11 for neonatal mortality 2011, child11 for child mortality 2011, neo14 for neonatal mortality 2014 and child14 for child mortality 2014. Number in bolds indicates the highest and lowest percentages. Additionally, all percentages described are sub-percentages of the overall neonatal and child mortality percentages for 2011 and 2014. For example, the overall neonatal mortality rate for 2011 is 5.78% [506/ (506 + 8247)]. The features of **Table 1** are discussed in Appendix S3 in **Online Supplementary Document(Online Supplementary Document)** while the graphical representations are in Figures S1-S16 in **Online Supplementary Document(Online Supplementary Document)**.

**Table 1:** Background characteristics of neonatal and child mortality in 2011 and 2014*

Study number	Covariates	2011				2014			
**Neonatal death**		**Child death**		**Neonatal death**		**Child death**	
**Total alive (8247)**	**Total death (506)**	**Total alive (8529)**	**Total death (224)**	**Total alive (7646)**	**Total death (240)**	**Total alive (7567)**	**Total death (319)**
1	**Region**								
	Barisal	927 (11.24)	50 (9.88)	947 (11.10)	30 (13.40)	887 (11.60)	**19 (7.90)**	882 (11.70)	**24 (7.50)**
	Chittagong	1665 (20.18)	85 (16.79)	1694 (19.90)	**56 (25.00)**	1474 (19.30)	43 (17.90)	1453 (19.20)	64 (20.10)
	Dhaka	1358 (16.46)	87 (17.19)	1415 (16.60)	30 (13.40)	1346 (17.60)	32 (13.30)	1335 (17.60)	43 (13.50)
	Khulna	938 (11.37)	**44 (8.69)**	970 (11.40)	**12 (5.40)**	829 (10.80)	33 (13.80)	822 (10.90)	40 (12.50)
	Rajshahi	1013 (12.28)	70 (13.83)	1054 (12.40)	29 (12.90)	927 (12.10)	32 (13.30)	923 (12.20)	36 (11.30)
	Rangpur	1047 (12.69)	60 (11.85)	1088 (12.80)	19 (8.50)	930 (12.20)	28 (11.70)	920 (12.20)	38 (11.9)
	Sylhet	1299 (15.75)	**110 (21.73)**	1361 (16.00)	48 (21.40)	1253 (16.40)	**53 (22.10)**	1232 (16.30)	**74 (23.20)**
2	**Type of place:**								
	Urban	2520 (30.55)	**154 (30.43)**	2614 (30.60)	**60 (26.80)**	2418 (31.60)	**70 (29.20)**	2399 (31.70)	**89 (27.90)**
	Rural	5727 (69.44)	**352 (69.56)**	5915 (69.40)	**164 (73.20)**	5228 (68.40)	**170 (70.80)**	5915 (69.40)	**230 (72.10)**
3	**Gender of child:**								
	Male	4214 (51.10)	**288 (56.9)**	4399 (51.60)	**103 (46.00)**	3927 (51.40)	**134 (55.80)**	3892 (51.40)	**169 (53.00)**
	Female	4033 (48.90)	**218 (43.10)**	4130 (48.40)	**121 (54.00)**	3719 (48.60)	**106 (44.20)**	3675 (48.6)	**121 (47.00)**
4	**Mother’s education:**								
	No education	1587 (19.20)	100 (19.76)	1607 (18.80)	62 (27.70)	1190 (15.60)	43 (17.90)	1169 (15.40)	64 (20.10)
	Primary	2506 (30.40)	178 (35.20)	2605 (30.50)	79 (35.30)	2133 (27.90)	73 (30.40)	2107 (27.80)	99 (31.00)
	Secondary	3482 (42.20)	**208 (41.10)**	3628 (42.50)	**80 (35.70)**	3509 (45.90)	**112 (46.70)**	3480 (46.00)	**141 (44.20)**
	Higher	672 (8.10)	**20 (3.95)**	689 (8.10)	**3 (1.30)**	814 (10.60)	**12 (5.00)**	811 (10.70)	**15 (4.70)**
5	**Father’s education:**								
	No education	2283 (27.70)	**179 (31.00)**	2374 (27.80)	**88 (39.30)**	1932 (25.30)	76 (31.70)	1902 (25.10)	**106 (33.20)**
	Primary	2396 (29.10)	157 (31.00)	2475 (29.00)	78 (34.80)	2298 (30.10)	**79 (32.90)**	2276 (30.10)	101 (31.70)
	Secondary	2432 (29.50)	129 (25.50)	2508 (29.40)	53 (23.70)	2291 (30.00)	69 (28.80)	2270 (30.00)	90 (28.20)
	Higher	1136 (13.80)	**41 (8.10)**	1172 (13.70)	**5 (2.20)**	1125 (14.70)	**16 (6.70)**	1119 (14.80)	**22 (6.90)**
6	**Mother’s occupation:**								
	Working	544 (6.60)	**37 (7.30)**	558 (6.50)	**23 (10.30)**	952 (12.50)	**29 (12.10)**	942 (12.40)	**39 (12.20)**
	Not working	7703 (93.40)	**469 (92.70)**	7971 (93.50)	**201 (89.70)**	6694 (87.50)	**211 (87.90)**	6625 (87.60)	**280 (87.80)**
7	**Father’s occupation:**								
	Farmer	3438 (42.10)	**221 (43.70)**	3588 (42.10)	**101 (45.10)**	1879 (24.60)	68 (28.30)	1854 (24.50)	93 (29.20)
	Business	3434 (41.60)	192 (37.90)	3550 (41.60)	76 (33.90)	4178 (54.60)	**123 (51.20)**	4144 (54.80)	**157 (49.20)**
	Service	505 (6.10)	**22 (9.30)**	519 (6.10)	**8 (3.60)**	501 (6.60)	**8 (3.30)**	498 (6.60)	**11 (3.40)**
	Others	840 (10.20)	71 (14.00)	872 (10.20)	39 (17.40)	1088 (14.20)	41 (17.10)	1071(14.20)	58 (18.20)
8	**Radio:**								
	No	7653 (92.80)	**473 (93.50)**	7919 (92.80)	**207 (92.40)**	6757 (88.40)	**222 (92.50)**	6684 (88.30)	**295 (92.50)**
	Yes	594 (7.20)	**33 (6.50)**	610 (7.20)	**17 (7.60)**	889 (11.60)	**18 (7.50)**	883 (11.70)	**24 (7.50)**
9	**TV**								
	No	5264 (63.80)	**352 (69.60)**	5447 (63.90)	**169 (75.40)**	4027 (52.70)	**151 (62.90)**	3977 (52.60)	**201 (63.00)**
	Yes	2983 (36.20)	**154 (30.40)**	3082 (36.10)	**55 (24.60)**	3619 (47.30)	**89 (37.10)**	3590 (47.40)	**118 (37.00)**
10	**Religion:**								
	Non-Muslim	802 (9.70)	**47 (9.30)**	826 (9.70)	**23 (10.30)**	7021 (91.80)	**12 (5.00)**	6953 (91.90)	**23 (7.20)**
	Muslim	7445 (90.30)	**459 (90.70)**	7703 (90.30)	**201 (89.70)**	625 (8.20)	**228 (95.00)**	614 (8.10)	**296 (92.80)**
11	**Wealth index:**								
	Poor	3408 (41.30)	**238 (47.00)**	3511 (41.20)	**135 (60.30)**	4618 (60.40)	**150 (62.50)**	4561 (60.30)	**207 (64.90)**
	Middle	1565 (19.00)	**96 (19.00)**	1627 (19.10)	**34 (15.20)**	1470 (19.20)	46 (19.20)	1460 (19.30)	56 (17.70)
	Rich	3274 (39.70)	172 (34.00)	3391 (39.80)	55 (24.60)	1558 (20.40)	**44 (18.30)**	1546 (20.40)	**55 (17.50)**
12	**SBI (months):**								
	<24	7478 (90.70)	**442 (87.40)**	7738 (90.70)	**182 (81.20)**	613 (8.00)	**181 (75.40)**	602 (8.00)	**230 (72.10)**
	25-48	693 (8.40)	60 (11.90)	712 (8.30)	41 (18.30)	1533 (20.00)	41 (17.10)	1514 (20.00)	60 (18.80)
	49 and above	76 (0.90)	**4 (0.80)**	79 (0.90)	**1 (0.40)**	5500 (71.90)	**18 (7.50)**	5451 (72.00)	**29 (9.10)**
13	**Birth order (years):**								
	One	7478 (90.70)	**442 (87.40)**	3079 (36.10)	43 (19.20)	2984 (39.00)	**110 (45.80)**	2960 (39.10)	134 (42.00)
	2-6	693 (8.40)	60 (11.90)	5253 (61.60)	**171 (76.30)**	4524 (59.20)	124 (51.70)	4470 (59.10)	**178 (55.80)**
	7 and above	76 (0.90)	**4 (0.80)**	197 (2.30)	**10 (4.50)**	138 (1.80)	**6 (2.50)**	137 (1.80)	**7 (2.20)**
14	**Type of toilet facility:**								
	Unhygienic toilet	6546 (79.37)	**382 (75.49)**	6758(81.94)	**170 (75.89)**	5572 (72.90)	**184 (76.70)**	5506 (72.80)	**250 (78.40)**
	Hygienic toilet	1701 (20.26)	**124 (24.50)**	1771 (21.47)	**54 (24.10)**	2074 (27.10)	**56 (23.30)**	2061 (27.20)	**69 (21.60)**
15	**Diarrhea:**								
	Yes	7861 (95.30)	**497 (98.20)**	8140 (95.40)	**218 (97.30)**	7170 (93.80)	**12 (95.00)**	7170 (94.80)	**10 (96.86)**
	No	386 (4.70)	**9 (1.80)**	389 (4.60)	**6 (2.70)**	476 (6.20)	**240 (5.00)**	397 (5.20)	**309 (3.14)**
16	**Mother’s age (years):**								
	15-24	4028 (48.80)	**315 (62.30)**	4223 (49.50)	**120 (53.60)**	3731 (48.80)	**129 (53.80)**	3698 (48.90)	**162 (50.80)**
	25-34	3486 (42.30)	158 (31.20)	3565 (41.80)	79 (35.30)	3296 (43.10)	91 (37.90)	3266 (43.20)	121 (37.90)
	35-44	699 (8.50)	29 (5.70)	704 (8.30)	37 (16.51)	582 (7.60)	18 (7.50)	567 (7.50)	33 (10.30)
	45 and above	34 (0.40)	**4 (0.80)**	24 (10.70)	**1 (0.44)**	37 (0.50)	**2 (0.80)**	36 (0.50)	**3 (0.90)**

SBI – succeeding birth interval

*Source: BDHS, 2011 and 2014. Numbers in bold indicate the highest and lowest percentages.

### Graphical interpretation of BDHS data: neonatal and child mortality

Figure S1(a) in **Online Supplementary Document(Online Supplementary Document)** shows that the relative order of the regions in terms of their neonatal mortality percentages changed significantly, but the three regions that increased their percentages (Chittagong, Sylhet, and Khulna) were the ones with the highest percentages in 2014. These three areas have some common criteria. Because, there are many rootless people live in this area and government intervention does not reach these areas properly. The increasing rate of neonatal mortality in 2014 than 2011 is ocular proof of delay medical intervention or negligence of medical worker or may be lake of health worker. Figure S1(b) in **Online Supplementary Document(Online Supplementary Document)** shows the same trends as Figure S1(a), with the exception of Rangpur, Dhaka, and Chittagong, which all had very large reductions in child mortality from 2011 to 2014, a pattern not visible in their neonatal mortality percentages. The steps taken in these regions should be applied to the entirety of Bangladesh to result in similar reductions.

Figure S2(a) and Figure S2(b) in **Online Supplementary Document(Online Supplementary Document)** indicate neonatal and child mortality are slightly high in urban in 2014 compared to 2011. But both mortalities are low in rural in 2014 than 2011. Clearly, the advancements available to urban families are necessary to reduce neonatal and child mortality in rural areas. Figure S3(a) and Figure S3(b) in **Online Supplementary Document(Online Supplementary Document)** show a startlingly high percentage for both males and females, with little change in neonatal mortality between 2011 and 2014. However, child mortality for males and females show a more discernible change, with the male percentage increasing over time and the female percentage decreasing during the same time. Figure S4(a) and Figure S4(b) in **Online Supplementary Document(Online Supplementary Document)** show that neonatal and child mortalities both increase most significantly at the level of secondary education from 2011 to 2014. Given that this increase is also present at the level of higher education, it seems that mothers beyond a primary level of education are not being targeted for contraception use with the assumption that they understand their own situation. Therefore, more attention needs to be given at this level in order to reduce child and neonatal mortality as a whole. Figure S5(a) in **Online Supplementary Document(Online Supplementary Document)** demonstrates that the only case in which neonatal mortality has decreased when the father has achieved a level of higher education. On the other hand, Figure S5(b) in **Online Supplementary Document(Online Supplementary Document)** presents a conflicting view that child mortality actually increases when the father has secondary or higher education between 2011 and 2014. Figure S6(a) and Figure S6(b) in **Online Supplementary Document(Online Supplementary Document)** represents that the mortality percentage increases between 2011 and 2014 is in the area of neonatal mortality with working mothers.

Maternity Benefit is a payment on maternity leave women from work. This benefit can decrease neonatal mortality by decreasing working pressure. It gives a family financial support. Paternal benefits or at least paternity leave is also essential for decreasing neonatal mortality rate. A neonatal needs intensive care when a mother has a tension about her job it not possible take care of her baby. Maternity benefits can be made her free of job tension and financial support. One can think maternal benefits may be a good solution to decrease the neonatal and child mortality. Figure S7(a) and Figure S7(b) in **Online Supplementary Document(Online Supplementary Document)** appear to be most homologous out of the seven covariates observed thus far. Figure S8(a) and Figure S8(b) in **Online Supplementary Document(Online Supplementary Document)**are even more similar than their seven counterparts. It is interesting to note that the presence of a radio is indicative of such a large disparity within the same regions.

Figure S9(a) and Figure S9(b) in **Online Supplementary Document(Online Supplementary Document)** show both mortalities are higher for whose families have TV in 2014 than 2011. In 2014, neonatal death and child deaths are higher in Muslim families compared to non-Muslim family (Figure S10(a) and Figure 10(b) in **Online Supplementary Document(Online Supplementary Document)**. This is consistent with the majority of the country being Muslim; therefore the majority of deaths will occur in Muslims. From 2011 to 2014, both mortalities are up in poor families because they do not get proper nutrition’s and health care facilities (Figure S11(a) and Figure S11(b) in **Online Supplementary Document(Online Supplementary Document)**. In middle-class families both mortalities are comparable in 2011 and 2014. But in rich families both mortalities are down in 2014 compared to 2011. In Figure S12(a) and Figure S12(b) in **Online Supplementary Document(Online Supplementary Document)**, as time between births increases, the likelihood of neonatal and child mortality decreases. The only case which seems to defy expectation is that of SBI 49 and above, where child and neonatal mortality both increases from 2011 to 2014. This may be because of the increased burden of having one or more children already 49 months or above of age and having to care for the other child on the way. We observe that from Figure S13(a) and Figure S13(b) in **Online Supplementary Document(Online Supplementary Document)** neonatal death is down and child death is up for first birth order in 2014 compared to 2011.

It is also observed for 2nd order neonatal death is slightly high and child death is down in 2014. Neonatal and child death is almost comparable for type of toilet facility in both years (Figure S14(a) and Figure S14(b) in **Online Supplementary Document(Online Supplementary Document)**. Figure S15(a) and Figure S15(b) in **Online Supplementary Document(Online Supplementary Document)** confirm our understanding that diarrhea is one of the most serious problems to address in Bangladesh. Figure S16(a) and Figure S16(b) in **Online Supplementary Document(Online Supplementary Document)** indicate that neonatal and child mortalities are down for mothers who are 15-24 years; whereas, both mortalities are up for mothers who are 25-34 years. Therefore, as these two categories produce the vast amount of the neonatal and child mortality in Bangladesh, increased incentives for the education of the youth in Bangladesh need to be put into action to lower these numbers.

### Segmentation of potential risk factors

Mosley et al [9] showed some factors influencing child survival in developing countries. The outcome variables were studied against all selected potential risk variables and these variables were divided into three distinct groups: (i) community, (ii) household and (iii) individual factors. (i) The community level factors are type of place and the geographical region. The type of place is divided into two categories (urban and rural) and geographic regions are divided into seven categories (Barisal, Chittagong, Dhaka, Khulna, Rajshahi, Rangpur, and Sylhet). (ii) The household factor was the wealth index variable that measured the financial status of a household. The household wealth index variable is divided into five categories (poorest, poorer, middle, richer and richest), but for our calculation, this index was divided into three categories (poor, middle and rich). The bottom 40% of households are of poor, the next 40% are of the middle and the top 20% are of the rich category households. (iii) The individual factors were: religion, watches TV, listens to radio, gender of the child, birth order, SBI, and paternal education.

### Methodological approach

Our methodology is based on application of bivariate analysis, namely χ-^2^ test, for examination of various predictors and response variables using a single population. We used the SPSS version 20.00 and R i386 version 3.4.1 software system for our analysis. The methodology used multiple logistic regression (MLR) where the goal was to determine which predictor variables influence on (a) neonatal mortality and (b) child mortality. The main predictor variables are: region, type of place, gender of child, mother’s and father’s education, mother’s and father’s occupation, radio, TV, religion, wealth index, SBI, birth order, type of toilet facility, diarrhea, and mother’s age.

**Figure 2:**
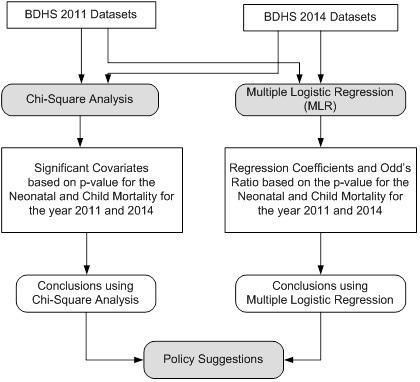
System block diagram.

The system block diagram has been shown in **Figure 2****.** It has two phases: BDHS 2011 data set (shown in left) and BDHS 2014 data set (shown in right side). χ^2^ analysis applied on both BDHS 2011 and 2014 data set. χ^2^-test gives only message as to which of the 16 covariates are associated or differed with mortality (neonatal and child). We determined significant covariates based on their *P*-values. Similarly, we also applied MLR on both data sets. MLR measures the average of the relationship between the categorical dependent variable (alive vs death) and two or more predictor variables (16 covariates).

χ^2^-test shows only the association between two categorical dependent variable (DV) and independent variables (categorical), but doesn't show the dependency of DV on independent variables. On the other hand, MLR shows both association and the dependency of DV on independent variables, hence can be adapted for studying the effect of change in independent variables on DV. **Figure 2** shows the system used for estimating the most significant covariates.

We use two strategies for understanding (a) the association between covariates and mortalities (neonatal and child) and (b) significance of the covariates when it comes to mortalities. For (a) we use chi-square test and for (b) we use multiple logistic regression (MLR). The corresponding mathematical expressions are given in Appendix S1 and S2 in **Online Supplementary Document(Online Supplementary Document),** respectively. Note that the *P*-value is the deciding criteria during the χ^2^-test analysis leading to the association between covariates and mortalities. On the contrary, the regression coefficients and the Odds ratio (OR) are adapted for concluding the significance of the covariates for mortalities.

## RESULTS

### Results using χ^2^-test: understanding associations

The χ^2^-test shows the association between the selected covariates of neonatal and child mortality. **Table 2** contains the results of chi-square test. The first column of the **Table 2** represents the attributes of selected covariates sequentially for which the association to be tested. Based on their respective *P*-values, in 2011, we say that the region, gender of child, father’s and mother’s education, father’s occupation, TV, wealth index, SBI, birth order, type of toilet facility, diarrhea and mother’s age are significant covariates for neonatal mortality. Whereas, region, father’s and mother’s education, father’s and mother’s occupation, TV, wealth index, SBI, birth order and mother’s age are also significant covariates for child mortality. In 2014, region, father’s and mother’s education, father occupation, radio, TV, birth order, diarrhea and mother’s age are significant factors for neonatal mortality, whereas, region, mother’s education, father’s and mother’s occupation, radio, TV, type of toilet facility, diarrhea and mother’s age are also significant factors for child mortality.

**Table 2:** Association of neonatal mortality and child mortality with respect to the selected covariates by χ^2^-test for 2011 and 2014*

Covariates	2011				2014			
**Neonatal mortality**		**Child mortality**		**Neonatal mortality**		**Child mortality**	
***-test***	***P*-value**	***-test***	***P*-value**	***-test***	***P*-value**	***-test***	***P* -value**
Region	**18.852**	***P* < 0.010**	**19.573**	***P* < 0.010**	**12.087**	***P* < 0.050**	**17.556**	***P* < 0.050**
Type of place	0.003	0.954	1.535	0.215	0.651	0.420	2.051	0.152
Gender of child	**6.464**	***P* < 0.050**	2.735	0.098	1.864	0.172	0.292	0.589
Mother’s education	**24.675**	***P* < 0.001**	**57.599**	***P* < 0.001**	**8.465**	***P* < 0.050**	**16.069**	***P* < 0.010**
Father’s education	**24.675**	***P* < 0.001**	**4.888**	***P* < 0.001**	**14.902**	***P* < 0.001**	0.014	0.906
Mother’s occupation	0.394	0.530	**16.714**	***P* < 0.010**	0.029	0.865	**21.269**	***P* < 0.001**
Father’s occupation	**11.142**	***P* < 0.050**	0.063	***P* < 0.010**	**6.882**	***P* < 0.050**	**12.535**	***P* < 0.001**
Radio	0.332	0.564	**12.733**	0.564	**3.894**	***P* < 0.050**	**5.168**	***P* < 0.001**
TV	**6.821**	***P* < 0.010**	0.085	***P* < 0.001**	**9.811**	***P* < 0.001**	**13.424**	***P* < 0.001**
Religion	0.104	0.798	**33.685**	0.771	3.158	0.075	0.337	0.562
Wealth index	**7.286**	***P* < 0.050**	**27.863**	***P* < 0.001**	0.649	0.723	2.806	0.246
SBI (months)	**9.316**	***P* < 0.010**	**29.433**	***P* < 0.001**	1.496	0.473	0.714	0.700
Birth order (years)	**10.872**	***P* < 0.010**	2.356	***P* < 0.001**	**5.585**	***P* < 0.050**	1.485	0.483
Type of toilet facility	**8.197**	***P* < 0.050**	1.795	0.502	1.697	0.193	**4.881**	***P* < 0.050**
Diarrhea	**9.316**	***P* < 0.001**	**37.139**	0.180	**27.478**	***P* < 0.001**	**29.123**	***P* < 0.001**
Mother’s age (years)	**37.139**	***P* < 0.001**		***P* < 0.001**	**3.205**	***P* < 0.001**	**6.787**	***P* < 0.001**

SBI – succeeding birth interval

*Source: BDHS, 2011 and 2014. Numbers in bold indicate significant covariates at 1%, 5% and 0.1%.

### Results using multiple logistic regression

We create **Table 3** and **Table 4** to show the logistics effect of the selected covariates on neonatal and child mortality. The columns of the **Table 3** and **Table 4** have been presented by selecting those covariates, regression coefficient (β), and odds ratios (OR) which are statistically significant. This constitutes only covariates such as: region, gender of child, mother’s and father’s education, father’s occupation, religion, SBI, birth order, type of toilet facility, diarrhea and mother’s age are statistically significant at 1, 5 and 10% level of significance. Rests of the covariates (type of place, mother’s occupation, radio, TV, wealth index) are not significant of that level of significance. Because their *P*-values are greater than 0.010, 0.050 and 0.001, so they have no effect on neonatal and child mortality. Here, we have discussed only the significant variable covariates which are given in **Table 3**. The description of the observations corresponding to **Table 3** and **Table 4** are shown in Appendix S4 and Appendix S5 in **Online Supplementary Document(Online Supplementary Document),** respectively. The importance of the 16 covariates in terms of its risk is presented in **Table 5**(columns 4 and 5).

**Table 3:** Multiple logistic regression estimates for the effect of the selected covariates on neonatal mortality in 2011 and 2014*

Study Number	Covariates	2011					2014				
**Coef. **	***P*-value**	**OR**	**95% CI for OR**		**Coef. **	***P*-value**	**OR**	**95% CI for OR**	
			**Lower**	**Upper**				**Lower**	**Upper**
1	**Region:**										
	Barisal (Ref)	**-**	**-**	1.000	-	-	**-**	-	1.000	-	-
	Chittagong	-0.093	0.620	0.911	0.632	1.315	0.122	0.711	1.130	0.592	2.159
	Dhaka	0.133	0.477	1.142	0.792	1.648	0.153	0.666	1.165	0.582	2.332
	Khulna	-0.127	0.555	0.881	0.577	1.343	0.914	0.015	2.494	1.196	5.202
	Rajshahi	0.249	0.201	1.282	0.876	1.877	**0.806**	***P* < 0.010**	**2.238**	**1.098**	**4.563**
	Rangpur	-0.036	0.858	0.965	0.650	1.431	**1.040**	***P* < 0.001**	**2.829**	**1.342**	**5.966**
	Sylhet	**0.416**	***P* < 0.050**	**1.517**	**1.060**	**2.171**	**0.649**	***P* < 0.050**	**1.914**	**0.996**	**3.679**
2	**Type of place:**										
	Urban (Ref)	**-**	-	1.000	-	-	**-**	-	1.000	-	-
	Rural	**-**0.172	0.134	0.842	0.673	1.054	-0.176	0.407	0.838	0.553	1.272
3	**Gender of the child:**										
	Male (Ref)	-	-	1.000	**-**	**-**	**-**	-	1.000	-	-
	Female	**0.275**	***P* < 0.010**	**0.759**	**0.632**	**0.968**	-0.080	0.634	0.923	0.663	1.285
4	**Mother’s education:**										
	No education (Ref)	-	-	1.000	-	-	-	-	1.000	-	-
	Primary	0.190	0.174	1.209	0.920	1.590	-0.118	0.648	0.888	0.535	1.476
	Secondary	0.239	0.128	1.270	0.930	1.726	0.259	0.348	1.295	0.755	2.223
	Higher	**0.054**	***P*0.001**	**1.056**	**0.575**	**1.937**	-0.133	0.789	0.876	0.332	2.311
5	**Father’s education:**										
	No education (Ref)	-	-	1.000	-	-	-	-	1.000	-	-
	Primary	-0.216	0.080	0.806	0.633	1.026	-0.079	0.724	0.924	0.598	1.428
	Secondary	**-0.350**	***P* < 0.050**	**0.705**	**0.532**	**0.935**	-0.113	0.656	0.893	0.542	1.470
	Higher	**-0.642**	***P* < 0.010**	**0.526**	**0.324**	**0.853**	**-0.910**	***P* < 0.050**	**0.403**	**0.163**	**0.992**
6	**Mother’s occupation:**										
	Working (Ref)	-	-	1.000	-	-	-	-	1.000	-	-
	Not working	-0.205	-0.264	0.814	0.568	1.168	0.032	0.902	1.033	0.618	1.724
7	**Father’s occupation:**										
	Farmer (Ref)	-	-	1.000	-	-	-	-	1.000	-	-
	Business	0.007	0.952	1.007	0.808	1.255	-0.402	0.068	0.669	0.435	1.030
	Service	**0.281**	***P* < 0.010**	**1.324**	**0.758**	**2.314**	0.411	0.499	1.509	0.458	4.970
	Others	0.239	0.101	1.270	0.954	1.692	-0.499	0.060	0.607	0.361	1.021
8	**Radio:**										
	No (Ref)	-	-	1.000	-	-	-	-	1.000	-	-
	Yes	0.030	0.873	1.031	0.710	1.496	-0.567	0.093	0.567	0.293	1.099
9	**TV:**										
	No (Ref)	-	-	1.000	-	-	-	-	1.000	-	-
	Yes	-0.175	0.178	0.840	0.651	1.083	-0.273	0.221	0.761	0.491	1.179
10	**Religion:**										
	Non-Muslim (Ref)	-	-	1.000	-	-	-	**-**	1.000	-	-
	Muslim	-0.070	0.669	0.932	0.676	1.285	**0.070**	***P* < 0.050**	**0.440**	**0.218**	**0.889**
11	**Wealth index:**										
	Poor (Ref)	-	-	1.000	-	-	-	-	1.000	-	-
	Middle	0.040	0.772	1.041	0.794	1.365	0.280	0.226	1.323	0.841	2.080
	Rich	0.034	0.830	1.034	0.760	1.408	0.231	0.337	1.260	0.786	2.018
12	**SBI:**										
	<24 (Ref)	-	-	1.000	**-**	-	-	-	1.000	-	-
	25-48	**0.353**	***P* < 0.010**	**1.423**	**1.064**	**1.903**	-0.107	0.763	.899	0.450	1.795
	49 and above	0.022	0.967	1.022	0.368	2.835	0.166	0.628	1.181	0.602	2.316
13	**Birth order:**										
	One (Ref)	-	-	1.000	**-**		-	-	1.000	-	-
	2-6	**0.744**	***P* < 0.001**	**2.105**	**1.677**	**2.642**	-0.266	0.286	0.767	0.470	1.249
	7 and above	**0.954**	***P* < 0.001**	**2.597**	**1.300**	**5.186**	0.167	0.812	1.182	0.299	4.674
14	**Type of toilet facility:**										
	Unhygienic toilet (Ref)	-	**-**	1.000			**-**		1.000		
	Hygienic toilet	**0.240**	***P* < 0.050**	**1.271**	**1.027**	**1.574**	0.144	0.549	1.155	0.721	1.851
15	**Diarrhea:**										
	No (Ref)	-	-	1.000	**-**	**-**	-	-	1.000	**-**	**-**
	Yes	**1.130**	***P* < 0.010**	**0.323**	**0.165**	**0.632**	**1.130**	***P* < 0.010**	**0.323**	**0.165**	**0.632**
16	**Mother’s age:**										
	15-24 (Ref)	-	-	1.000	**-**	**-**	-	-	1.000	**-**	**-**
	25-34	**-0.899**	***P* < 0.001**	**0.407**	**0.326**	**0.508**	**0.036**	***P* < 0.001**	**0.407**	**0.326**	**0.508**
	35-44	**-1.047**	***P* < 0.001**	**0.351**	**0.227**	**0.542**	**0.015**	***P* < 0.001**	**0.351**	**0.227**	**0.542**
	45 and above	-0.162	0.779	0.851	0.275	0.634	-0.054	0.779	0.851	0.275	0.634

OR – Odds ratio, Coef – Coefficients, CI – Confidence interval, Ref – Reference category

*Source: BDHS, 2011 and 2014. Numbers in bold indicate significant covariates at 1%, 5% and 0.1%.

**Table 4:** Multiple logistic regression estimates for the effect of the selected covariates on child mortality in 2011 and 2014*

Study Number	Covariates	2011					2014				
**Coef. **	***P*-value**	**OR**	**95% CI for OR**		**Coef. **	***P*-value**	**OR**	**95% CI for OR**	
			**Lower**	**Upper**				**Lower**	**Upper**
1	**Region:**										
	Barisal (Ref)	-	-	1.000	-	-	-	-	1.000	-	-
	Chittagong	-0.017	0.943	0.983	0.617	1.567	-0.469	0.125	0.625	0.344	1.138
	Dhaka	-0.450	0.095	0.638	0.376	1.081	-0.369	0.264	0.691	0.362	1.322
	Khulna	**-0.744**	***P* < 0.050**	**0.475**	**0.239**	**0.943**	**-1.074**	***P* < 0.010**	**0.342**	**0.168**	**0.694**
	Rajshahi	-0.161	0.551	0.851	0.501	1.446	**-0.758**	***P* < 0.050**	**0.469**	**0.236**	**0.929**
	Rangpur	**-0.819**	***P* < 0.010**	**0.441**	**0.243**	**0.802**	**-1.342**	***P* < 0.001**	**0.261**	**0.126**	**0.541**
	Sylhet	-0.115	0.644	0.891	0.547	1.452	**-0.872**	***P* < 0.001**	**0.418**	**0.226**	**0.774**
2	**Type of place:**										
	Urban (Ref)	-	-	1.000	-	-	-	-	1.000	-	-
	Rural	-0.200	0.253	0.818	0.580	1.154	-0.085	0.675	1.089	0.732	1.620
3	**Gender of child**										
	Male (Ref)	-	-	1.000	-	-	-	-	1.000	-	-
	Female	0.198	0.152	1.219	0.930	1.599	-0.101	0.531	0.904	0.660	1.239
4	**Mother’s education:-**										
	No education (Ref)	-	-	1.000	-	-	**-**	-	1.000	-	-
	Primary	**-0.454**	***P* < 0.010**	**0.635**	**0.448**	**0.900**	0.232	0.346	1.261	0.779	2.042
	Secondary	**-0.697**	***P* < 0.001**	**0.498**	**0.325**	**0.762**	-0.095	0.718	0.909	0.542	1.524
	Higher	-1.179	0.080	0.307	0.082	1.150	0.500	0.287	1.649	0.656	4.146
5	**Father’s education:**										
	No education (Ref)	-	-	1.000	-	-	-	-	1.000	-	-
	Primary	0.099	0.287	1.203	0.856	1.691	0.166	0.436	1.181	0.778	1.793
	Secondary	1.396	0.701	1.087	0.710	1.663	0.070	0.773	1.073	0.666	1.729
	Higher	**-0.355**	***P* < 0.010**	**0.187**	**0.059**	**0.595**	**0.770**	***P* < 0.050**	**2.160**	**0.950**	**4.914**
6	**Mother’s occupation:**										
	Working (Ref	-	-	1.000	-	-	-	-	1.000	-	-
	Not working	-0.502	0.034	0.605	0.380	0.963	-0.108	0.664	0.897	0.550	1.463
7	**Father’s occupation:**										
	Farmer (Ref)	-	-	1.000	-	-	-	-	1.000	-	-
	Business	0.097	0.564	1.102	0.792	1.534	**0.533**	***P* < 0.010**	**1.704**	**1.122**	**2.588**
	Service	1.388	0.324	4.008	1.645	9.763	-0.360	0.525	0.697	0.229	2.121
	Others	0.355	0.075	1.426	0.965	2.107	**0.462**	***P* < 0.050**	**1.587**	**0.963**	**2.614**
8	**Radio:**										
	No (Ref)	-	-	1.000	-	-	-	-	1.000	-	-
	Yes	0.379	0.155	1.460	0.867	2.460	0.494	0.112	1.639	0.890	3.016
9	**TV:**										
	No (Ref)	-	-	1.000	-	-	-	-	1.000	-	-
	Yes	0.028	0.897	1.028	0.676	1.564	0.094	0.659	1.099	0.723	1.671
10	**Religion:**										
	Non-Muslim (Ref)	-	-	1.000	-	-	-	-	1.000	-	-
	Muslim	-0.269	0.253	0.764	0.482	1.212	0.272	0.379	1.313	0.716	2.407
11	**Wealth index:**										
	Poor (Ref)	-	-	1.000	-	-	-	-	1.000	-	-
	Middle	-0.404	0.063	0.668	0.436	1.023	-0.099	0.655	0.905	0.585	1.401
	Rich	-0.521	0.037	0.594	0.365	0.968	-0.176	0.445	0.839	0.534	1.317
12	**SBI:**										
	<24 (Ref)	-	-	1.000	-	-	-	-	1.000	-	-
	25-48	**0.804**	***P* < 0.001**	**2.235**	**1.555**	**3.213**	0.286	0.380	1.332	0.703	2.522
	49 and above	-0.634	0.533	0.531	0.072	3.888	0.242	0.450	1.273	0.681	2.382
13	**Birth order:**										
	One (Ref)	-	-	1.000	**-**	**-**	-	-	1.000	-	-
	2-6	**1.042**	***P* < 0.010**	**2.835**	**1.941**	**4.140**	0.280	0.237	1.324	0.832	2.107
	7 and above	**1.285**	***P* < 0.050**	**3.614**	**1.563**	**8.356**	0.833	0.228	2.301	0.593	8.933
14	**Type of toilet facility:**										
	Unhygienic toilet (Ref)	-	-	1.000	-	-	-	-	1.000	-	-
	Hygienic toilet	0.163	0.315	1.177	0.857	1.617	0.099	0.667	1.104	0.704	1.732
15	**Diarrhea:**										
	No (Ref)	-	-	1.000	-	-	-	-	1.000	-	-
	Yes	-0.723	0.086	0.485	0.212	1.108	-0.723	0.086	0.485	0.212	1.108
16	**Mother’s age:**										
	15-24 (Ref)	-	-	1.000	-	-	-	-	1.000	-	-
	25-34	**-0.769**	***P* < 0.001**	**0.463**	**0.336**	**0.639**	-0.063	0.759	0.939	0.626	1.407
	35-44	**-0.586**	***P* < 0.050**	**0.557**	**0.331**	**0.937**	**-0.757**	***P* < 0.050**	**0.469**	**0.233**	**0.943**
	45 and above	-1.356	0.203	0.258	0.032	2.077	-0.979	0.339	0.376	0.050	2.801

OR - Odds ratio, Coef. – Coefficients, SBI – succeeding birth interval

*Source: BDHS, 2011 and 2014. Numbers in bold indicate significant covariates at 1%, 5% and 0.1%.

**Table 5:** Definition and categorization of the variables used in the analysis

Study Number	Name of variable	Definition and categorization	Importance of the Variable	
**Neonatal Mortality (Neo11 vs Neo14)**	**Child Mortality (Child11 vs Child14)**
1	Geographical region	There are seven regions of the country Bangladesh: (i) Barisal, (ii) Chittagong, (iii) Dhaka, (iv) Khulna, (v) Rajshahi, (vi) Rangpur and (vii) Sylhet	**Sylhet region** is **2nd risk factors** of neo11.**Rangpur region** is the highest risk factors for neo14.	Khulna region is lower risk for child11. Rajshahi region is the highest risk factor for child14.
2	Type of place	There are two type of place as: (i) rural and (ii) urban	It is not the risk factors for both neo11 and neo14.	It is not risk factors for both child11 and child14.
3	Gender of child	Gender considered for the child: male and female	Female child are lower risk for neo11 compared to male child. Female child has no risk for neo14.	Female child is not risk factors for both child11 and child14
4	Mother’s education	There are four categories of mother’s education as: (i) No education, (ii) Primary, (iii) Secondary, and (iv) Higher	**Mother’s education** is the **6th** risk factors of neo11.But it has no risk factors in neo14 (improve mother’s education).	Mother’s education is the **3rd risk factors** of child11. But it has no risk factors in child14. (Improve mother’s education)
5	Father’s education	There are four categories of father’s education as: (i) No education, (ii) Primary, (iii) Secondary, and (iv) Higher	**Father’s education** is an important risk factor for neo11 and neo14.	**Father’s education** also is an important risk factor (6th) for child11. **Father’s education** also is the **6th highest** risk factor for child14.
6	Mother’s occupation	There are two categories of mother’s occupation as: (i) Working and (ii) not working	It is not risk for both neo11 and neo14.	It is not risk for both child11 and child14.
7	Father’s occupation	There are four categories of mother’s occupation as: (i) Farmer, (ii) Business(iii) Service, and (iv) Others	**Father’s occupation** is the **top four highest risk** factors for neo11. It has no risk for neo14.	Father’s occupation has no risk factors for child11. It is the **2nd highest** risk factor for child14.
8	Radio	There are two categories of listening radio as: (i) No, and (ii) Yes	It has no risk for neo11 and neo14.	It has no risk for child11 and child14.
9	TV	There are two categories of watching radio as: (i) No, and (ii) Yes	It has no risk for neo11 and neo14.	It has no risk for child11 and child14.
10	Religion	There are two types of religion are used as: (i) Non-Muslim and (iii) Muslim	Religion has no risk for neo11**Religion** is the **2nd highest** risk factors for neo11.	Religion has no risk for child11 and child14.
11	Wealth index	There are 3 categories of wealth index as: (i) Poor (ii) Middle, (iii) Rich	Wealth index has no risk for neo11 and neo14.	Wealth index has no risk for child11 and child14.
12	Succeeding birth interval (SBI)	There are 3 categories of SBI as: (i)<24, (ii) 25-48(iii) 49 and above	**SBI** is the **3rd** risk factor for neo11. It has no risk for neo14.Improvement was noticed.	SBI is the **2nd risk factors** for child11It has no risk for child14.
13	Birth order (months)	There are 3 categories of birth order as: (i) One, (ii) 2-6, (iii) 7 and above	**Birth order** is the **top one** risk factor for Neo11. It has no risk for Neo14.	Birth order is also the top one risk factor for child11. It has no risk for child14.
14	Type of toilet facility	Two categories of type of toilet facility as: (i)Unhygienic toilet, and (ii) Hygienic toilet	It is the **top five** risk factors for neo11. It has no risk for neo14.	It is the **top two** risk factors for child11. It has no risk for child14.
15	Diarrhea	There are two categories who having Diarrhea as: (i) No and (ii) Yes	**Diarrhea** is the **top ten** risk factors for Neo 11.It is also the **top five** risk factors for Neo14.	It has no risk for both child11 and child14.
16	Mother’s age	There are 3 categories of Mother’s age as: (i) 15-24(ii) 25-34, (iii) 35-44, and (iv) 45 and above	**Mother’s age** is the **top nine** risk factors for Neo11.It is also the **top four** risks factors for Neo14.	**Mother’s age** is the **top four** risk factors for child11. It is also the **top three** risks factors for child14.

### Comparative study between 2011 and 2014

Using χ-^2^ test, we have got 12 significant covariates out of 16 covariates for neonatal mortality in 2011 and 9 covariates in 2014. This is shown in Appendix S6 in **Online Supplementary Document(Online Supplementary Document),** entitled “Plots of Most Significant Factors using χ^2^ for Neonatal and Child Mortality: 2011 vs. 2014”. In 2011, Figure S17(a) in **Online Supplementary Document(Online Supplementary Document)** indicates that first four top significant covariates are: mother’s age, mother’s education, father’s education and regions. Father’s occupations, birth order, diarrhea, SBI are the medium importance covariates and rests of the four covariates are lower importance. On the contrary, diarrhea and father’s education are the top covariates in 2014 (Figure S17(b) in **Online Supplementary Document(Online Supplementary Document)**) and these are the higher importance covariates. Using χ-^2^ test for child mortality, 10 significant covariates are in 2011 and 9 covariates in 2014. Figure S18(b) in **Online Supplementary Document(Online Supplementary Document)** indicates that mother’s education is the top significant covariates in 2011. The medium importance covariates are: mother’s age, father’s education, wealth index, birth order, and SBI and rests of the four covariates are lower importance. On the other way, Figure S18(b) in **Online Supplementary Document(Online Supplementary Document)** shows that diarrhea is the highest importance covariates in 2014. The medium importance covariates are mother’s occupations, region, mother’s education, TV, and father education, respectively. The bottom 3 covariates are: mother’s age, radio, and type of toilet facility. In 2011, using MLR, we have 10 significant covariates out of 16 covariates for neonatal mortality based on their *P*-value and odds ratio. We have arranged the odds ratio in descending order (from largest to smallest). Then we plot these significant covariates vs odds ratio. From Figure S19(a) in **Online Supplementary Document(Online Supplementary Document)**, we see that first top significant covariate is birth order and it is high importance covariates for neonatal mortality. The middle level covariates are: Sylhet region, SBI, father occupation, type of toilet facility, and mother’s education and these are medium importance covariates. The bottom 4 covariates are: gender of child, father’s education, mother’s age and diarrhea.

In 2014, we have only 5 significant covariates (Figure S19(b) in **Online Supplementary Document(Online Supplementary Document)**), so there was an improvement in socio-economic conditions. Among these 5 covariates, Rangpur region was the highest importance covariates. The rest of the importance covariates are religion, father education, mother’s age and diarrhea. In 2011, we have 6 significant covariates for child mortality (Figure S20(a) in **Online Supplementary Document(Online Supplementary Document)**. Among them birth order and SBI was the highest importance covariates for child mortality. The lower risk significant factors are: mother’s education, mother’s age, Khulna region, and father’s education, whereas, we have only 4 significant covariates out of 16 covariates in 2014. Father’s education and father’s occupation are highly importance factors for child mortality and the lowest importance factors are Rajshahi region and mother’s age (Figure S20(b) in in **Online Supplementary Document(Online Supplementary Document)**).

## PLAUSIBLE POLICY SOLUTIONS

### Primary solution

Mother’s education exerts an influence on both child survival and the management of childhood diseases. There is a positive relationship among the formal education of the mother, the use of prenatal care, and delivery assistance. This relationship has been shown to hold up even after controlling a range of indicators of income, social status, and access to health services. The reasoning for this comes from the increased consciousness from educated mothers about their own life and the life of their children. In most of the developing countries, children having a mother with secondary or higher education were at a lower risk of child mortality compared to children having a mother with no education [20]. Lalou et al [21] showed that there was a strong relationship between educating women and child life expectancy as well as progresses in child and family health and nutrition. There are five potential pathways linking maternal education and child health: (i) improved socio-economic status; (ii) health knowledge; (iii) modern attitudes towards health care; (iv) female autonomy; and (v) reproductive behavior. Investments in women’s education are important for lowering infant and child mortality. When children are born, the Ministry of Education of Bangladesh should message to families to begin their child’s schooling at the age of 5 years old [22]. Additionally, kids must be required to go to school up to age of 18 years, ie, grade 12 (high school). In the school, there should be a mandatory education about marriage age, priority for job, and challenges during early marriage and births. Those families who undergo education up to college must be discounted if they are living in rural areas. This will encourage more students go to college and prevent early marriages. Gender disparity in primary and secondary education must be eliminated to eradicate poverty and hunger, combat disease and ensure environmental sustainability.

Family planning is required to prevent as many as one in every three maternal deaths by allowing women to delay motherhood, space births, avoid unintended pregnancies and abortions, and stop childbearing when they have reached their desired family size [23]. After giving birth, family planning also help women wait at least two years before trying to become pregnant again, thereby reducing newborn, infant, and child deaths significantly [24]. Healthcare systems are an important organization in spreading the benefits of controlled births and the gap between the births of new born [25]. When the first kid is born, the doctors must spread messages to families to have the second child only after 3 years of age. Child mortality under five years is related to births spaced too close to one another, to large families, and to high birth orders. Better timing and spacing of pregnancies improves child health outcomes. As a result child mortality may be reduced. According to the WHO, about 80% of health care in developing countries occurs in the home and the majority of children who die do so at home, without being seen by a health worker. About 40% of child deaths may prevented with controlling family and community care not high-tech health equipment, but access to solid knowledge, support and basic supplies [26].

### Secondary solution

Participation in sports is also plausible solution of child mortality. It must be mandatory for kids to undergo sports curriculum and physical exercises and its benefits [27]. Teachers must be trained to talk about the advantages of marriages encouraged after 18 years or preferred after under-graduate years. The role of social media should be tapped in spreading information about college education, jobs by the government of Bangladesh [28]. Radios and Movie Theaters must be made by the government to put out messages about child birth, education, year’s gap between new born and jobs. We need to increase communication between urban and rural regions. In urban areas, kids get better services such as medical facility, education facility and so on. Government of Bangladesh must take the necessary steps to apply these same facilities to rural areas. The use of family planning can reduce infant mortality, by reducing the incidence of short birth intervals.

## DISCUSSION

### Analysis

Akter et al [14], Chowdhury et al [17] and Chowdhury et al [29] studied neonatal and child mortality in Bangladesh. However, they did not consider region as a significant factor (covariate). In this study, we show that regions is a significant influence on both neonatal and child mortalities for 2011 and 2014. In 2011, Sylhet region was higher risk of neonatal mortality. Whereas, in 2014, our findings showed that the risk of neonatal death was higher in three regions comparing to Barisal region. We order based on their odds ratio (OR) such as: (a) Rangpur (OR = 2.829), (b) Rajshahi (OR = 2.238) and (c) Sylhet (OR = 1.914). Because, a low number of prenatal care visits and low birth weights have been associated with post neonatal death [30]. Some of the regions in Bangladesh lack access to health facilities. As a result, there are regional differences in neonatal deaths. Most of the developing communities are more likely to get better sanitation connections that will improve child survival [31]. On the contrary, Khulna and Rangpur regions were lower risk of child mortality in 2011 and 2014. Moreover, the risk of child mortality was also lower in Rajshahi, and Sylhet compared to Barisal region in 2014.

Our findings show that male neonates and child’s had a significantly lower risk of dying during the neonatal and child’s period in 2011. On other way, this factor (gender of child) had improved in 2014. Mother’s education had a significantly higher risk of neonatal mortality and lower risk of child mortality in 2011. On the contrary, mother’s education was improved for the year of 2014. Father’s education had a great influence on the survival of young children. The previous studies showed that early childhood mortality was highly associated with the father education for all major developing countries. Chowdhury et al [29] showed father’s education was not a significant influence on both neonatal and child mortality. In our present study, father’s education has a significant influence on both neonatal and child mortality in 2011. In 2014, father’s education has also impact on both neonatal and child mortality. So we conclude that by improving father’s education, the childhood mortality rate will decrease. This is because an educated father is more conscious of their own life and children (see **Table 6**).

**Table 6:** Key difference between current study and previous study

Authors	Number of variables	Names of variables	Data size	BDHS	Methods	Highest important risk factors
Akter et al [14]	4	Mother’s education, Father’s education, Mother’s age, Place of residence	39875&38544	1995-2002&2002-2007	& MLR	Mother’s education
Chowdhury et al [29]	7	Mother’s education, Father’s education, Mother’s occupation, Father’s occupation, Wealth index, Types of toilet facilities, Electricity	796	No	& MLR	Mother’s education, Occupation, Type of latrine, Electricity
Chowdhury et al [17]	9	Mother’s education, Father’s education, Wealth index, Type of Place, Region, SBI, Child’s age, Mother’s age, Death of sibling	4003	2007	& MLR	Father’s education, Place of residence, Wealth, Index, Child’s age
Proposed Study (2017)	16	Region, Type of place, Gender of child, Mother’s education, Father’s education, Mother’s occupation, Father’s occupation, Radio, TV, Religion, Wealth index, SBI Birth order, Type of toilet facility, Diarrhea, Mother’s age	8753&7882	2011&2014	& MLR	NM (neo14) Region (Rangpur), Religion (Muslim), Father’s education, Mother’s Age, Diarrhea, CM (child14), Father’s education, Father’s occupation, Region (Rajshahi), Mother’s age, Improvement: Birth Order improved from 2011 to 2014 for neo11 and neo14.

BDHS – Bangladesh Demographic and Health Survey, MLR – multiple logistic regression

Father’s occupation is a powerful influence on neonatal and child mortality because he maintains an economic status, nutrition and housing condition, access to health care facilities, and clothing for the family. Father’s occupation is also associated with nutritional status of their children. The rate of neonatal and child mortality is higher for a business and others compared to service. Chowdhury et al [29] studied that father’s occupation was not a significant influence on neonatal mortality and child mortality. In this study we showed that father’s occupation had significant influence on neonatal mortality in 2011, whereas, father’s occupation had no effect on neonatal mortality in 2014. So it is say that this factor had improved for neonatal mortality in 2014. On the contrary, father’s occupation had no significant influence on child mortality in 2011, whereas, in 2014, it had significant higher risk of child mortality. In this study also showed that while father’s occupation are in farmer posed a risk to child mortality, father’s occupation are in business and others increased the odds of child mortality (see **Table 6**).

Religion is also very important factor in relation to mortality. In 2011, religion had no effect on both neonatal and child mortality, whereas, religion has significant effect on neonatal mortality in 2014. Our findings showed that Muslim children are at increased risk of neonatal death compared to non-Muslims. Particularly some Islamic people believe in myths concerning child birth. It is evident that Muslim women have higher fertility, lower rate of contraceptive use, early age at marriage and motherhood and lower access to maternal health care services utilization than their peer non-Muslim sisters in Bangladesh [32]. It is also evident that neonatal mortality has a positive influence on fertility. There is no doubt that Muslim women have higher fertility compared to non-Muslims. As a result the rate of neonatal death may be increased. Finally we conclude that traditional practices, cultural norms and religious faith are partly attributed to higher rate of neonatal death among the Muslims. SBI and birth order were the most significant covariates in 2011 were SBI between 25-48 months and birth order between 7 and above years having higher odds ratio as: 2.235 and 3.614. In 2014, SBI and birth order may be improved. Diarrhea showed the highest positive coefficient *P* < 0.01) leading to most significant covariate of neonatal mortality for both 2011 and 2014. The corresponding odds ratios were: 0.323 and 0.323 for both the years. Our findings also showed that there were lower risk of neonatal and child deaths in 25-34 and 35-44 age’s mother than 12-24 age of mother. Machado and Hill [6], Markovitz et al [33] and Seedhom and Kamal [34] showed a higher risk of neonatal mortality in younger adolescents than older mothers.

### Benchmarking

Using **Table 6**, we can see that Chowdhury et al [29] used the mortality data set of Natore sadar upzilla in Natore district of Bangladesh. This data was collected from 796 women. The factors selected for study were: parents’ education, parents’ occupation and wealth index. They used χ^2^-test to show how much the selected factors are associated with neonatal and child mortality. They also used logistic regression to show the dependency of selected factors on both mortalities. From the result of logistic regression, it was found that mother’s education, father’s occupation and wealth index were significant influencing factors on both neonatal mortality and child mortality. BDHS 2007 data set were used by Chowdhury et al [17]. The data set consisted of 4003 samples. They are also used both chi-square and logistic regression for analysis. They showed that father’s education, wealth index and birth order were risk factors for neonatal mortality.

Akter et al [14] used the two set mortality data that consisted of 39875 samples in 1995-2000 and 38544 samples in 2000-2007. They only showed the association between parents’ education and child mortality by using χ^2^-test and logistic regression. Their study result indicated that mother’s education was highly significant influence on child’s mortality. Uddin et al [35] used BDHS 1999-2000, that consisted of 6686 samples. They identified the potential risk factors were: mother’s and father’s education, mother’s and father’s occupation, birth order, mother’s age were responsible for neonatal and child mortality. However, in our study, χ^2^-test shows that mother’s and father’s education, mother’s and father’s occupation, wealth index, birth order, SBI and region are associated with both neonatal mortality and child mortality in 2014. On the contrary, MLR shows that Rangpur region, father’s education, religion, diarrhea and mother’s age are significant influence on neonatal mortality, whereas, region, father’s education and occupation, and mother’s age are also significant factors on child mortality.

### Limitations

We demonstrated the following itemized items in our study: (i) usage of the latest BDHS 2014 data sets; (ii) The study utilized first time 16 covariates compared to previous studies with limited covariance; (iii) Utilization of the χ^2^-test to show the association between neonatal and child mortality for 16 covariates on the basis of *P*-values; (iv) Utilization of MLR to identify the risk factors on the basis of odds ratio (OR) and *P*-values; (v) Besides the conventional risk factors, we showed new and first time introduced risk factors which are responsible for neonatal and child mortality, demonstrating difference compared to previous studies; (vi) Comprehensive analysis in understanding the factors which influence on the neonatal and child mortality over time; (vii) Suggests policy solutions based on the most significant covariates or risk factors to reduce neonatal and child mortality. Even though the system offered several novelties, it had some challenges.

First, the survey interviewing surviving women may be considered as a limitation, and this may have led to an under estimation of mortality rates because of the association between child and maternal deaths. The effect of some of the associated factors, such as delivery complications, could have also been underestimated. Second, most of the variables in this paper are not infant specific as they only reflected the most recent conditions or birth, like mother’s work status, which represented the employment status within the past 12 months preceding the survey. In future, we will study the multilevel modeling which takes into account the effect of clustering to better estimate the level of association of the study factors with the outcome.

### Extension of current work

Correspondence analysis (CA) may be used to show the association between mortalities and covariates instead of using chi-square criteria. To find the dependency of neonatal and child mortality on covariates, we may use another regressions such as multivariate proportional hazards models, step-wise regression, and multilevel logistic regression instead of multiple logistic regression. We will want to predict neonatal and child mortality by applying machine learning techniques such as Linear discriminant analysis (LDA), Quadratic discriminant analysis (QDA), Support vector machine (SVM), Gaussian process-based model (GP), Artificial neural network (ANN), Convolutional neural network (CNN), Fast neural network (FNN), and AdaBoost. We want to also monitoring the effect of covariates and mortalities changed over time.

## CONCLUSIONS

In 2011, using MLR, we got 10 significant covariates out of 16 covariates for neonatal mortality. These covariates are birth order, Sylhet region, SBI, father occupation, type of toilet facility, mother’s education, and gender of child, father’s education, mother’s age and diarrhea. In 2014, we got only 5 significant covariates, so there was an improvement in socio-economic conditions. In spite of improving socio-economic conditions, father’s education, mother’s age and diarrhea were still significant covariates for neonatal mortality in 2014. On the other hand, in 2011, we got 6 significant covariates for child mortality. Among them birth order and SBI was the highest importance covariates for child mortality. The lower risk significant factors were: mother’s education, mother’s age, Khulna region, and father’s education. In 2014, mother’s age and father’s education were also still significant covariates for child mortality. This study allows policy makers to make appropriate decisions to reduce neonatal and child mortality in Bangladesh.
