# Supplementary material for: Risk factors of neonatal mortality and child mortality in Bangladesh
Source: J Glob Health. 2018 Apr 30;8(1):010417. doi: 10.7189/jogh.08.010421 (PMC5928324; doi:10.7189/jogh.08.010421)
Supplement: Online Supplementary Document [file jogh-08-010421-s001.pdf]

Online Supplementary Document

Maniruzzaman et al. Risk factors of neonatal mortality and child mortality in Bangladesh

J Glob Health 2018;8:010421

Supplemental Appendices

Appendix A: Chi-Square Test

The standardized Chi-Squared test is given as:

$$\chi^2 = \sum_{i=1}^{r}\sum_{j=1}^{c}\frac{{O_{ij}}^2}{E_{ij}} - N \qquad (1)$$

Where, O_{ij} is the observed data for the location (i, j), E_{ij} is the expected data for location (i, j), r is the total number of rows; c is the total number of columns and N is the total number of observations. This follows a χ^2distribution with $(r-1)\times(c-1)$ degrees of freedom, respectively.

Appendix B: Multiple Logistics Regression (MLR)

In statistics, logistic regression is a form of regression model, which is used when the dependent variable (DV) (child and neonatal mortality) or response variable is categorical (having two values: death or alive) (Walker, S. H., and Duncan, D. B. 1967). Categorical in the sense that it has binary state having two values such as pass vs. fail, win vs. lose, alive vs. dead and so on.

Logit Model: A statistician David Cox developed logistic regression in 1958. The binary logistic model is used to estimate the probability of a binary response based on one or more predictor variables (features or covariates in our case). It also measures the relationship between the categorical dependent variable (alive vs. death of the child) and one or more predictor variables covariates) by estimating probabilities using a logistic function. Instead of Y as a function of X, we model logit of Y as function of linear combination of X (the covariates) and can be given as:

$$\text{logit}\,(P_j) = \log_e\left(\frac{P_j}{1-P_j}\right) = \sum_{i=0}^{K} \beta_i X_i$$

where, P_j is defined as the probability that Y=1 (child is alive) and 1-P_j (child is dead) is defined when Y=0. β_i's $(i = 0,1,..,K)$ are unknown constants (coefficients) to be determined from the data, K is the total number of predictor variables (16 covariates) and X_i $(i = 1,..,K)$ is the set of predictor variables and $X_0 = 1$. Note, Y dichotomous response variable for neonatal and child mortality and take value 1 and 0 that is Y is classified in the following way:

$$Y = \begin{cases} 1 & \text{if neonatal death occurs} \\ 0 & \text{otherwise} \end{cases} \qquad (2)$$

and

$$Y = \begin{cases} 1 & \text{if child death occurs} \\ 0 & \text{otherwise} \end{cases} \qquad (3)$$

The logit function logit (P_j) is the natural $\log_e$ of the odds that Y equals one of the two categories: alive or death of the child. It is not only linear in covariates (X_i) but also linear in parameters (β_i). We estimate the unknown regression coefficients β_i of logistic regression by applying maximum likelihood estimator (MLE) (Menard, Scott W (2002), Czepiel, S. A (2002)). It is not possible to find the simple expression for regression coefficients that maximize the likelihood function unlike linear regression. An iterative method is used as like Newton's method. These coefficients (β_i) tell you how much the logit changes based on the value of covariates (16 covariates, in our study). Positive β_i having low p-value for that covariate will show high significance and vice-versa. Since Y is two categorical (alive vs. death), it follows Bernoulli distribution. The probability mass function of Y is

$$f(Y|\beta) = P_j^{\,y}(1-P_j)^{1-y} \qquad ; y = 0, 1$$

The Principal of MLE is

- Write down the likelihood function. That means the product of the probability mass function. *i.e.,*

$$L(\beta|Y) = \prod_{r=1}^{n} f\,(y_r, \beta) = \prod_{r=1}^{n} P_j^{y_r}\left(1 - P_j\right)^{1-y_r}$$

$$= \prod_{r=1}^{n} \left(\frac{P_j}{1 - P_j}\right)^{y_r} \left(1 - P_j\right)$$

$$= \prod_{r=1}^{n} \left[\left(\exp\left(\sum_{i=0}^{K} \beta_i X_i\right)\right)^{y_r} \left(\frac{1}{1 + \exp(\sum_{i=0}^{K} \beta_i X_i)}\right)\right]$$

- Taking $\log_e$ on the likelihood function, then we have

$$l(\beta) = \log_e L(\beta|Y) = \sum_{r=1}^{n} y_r \left(\sum_{i=0}^{K} \beta_i X_i\right) - n\log_e\left(1 + \exp\left(\sum_{i=0}^{K} \beta_i X_i\right)\right)$$

- Differentiating of $l(\beta)$ with respect to β_i and setting equal to zero

$$\frac{\partial l(\beta)}{\partial \beta_i} = \sum_{r=1}^{n} y_r\, X_i - \frac{n\, X_i \exp\left(\sum_{i=0}^{K} \beta_i X_i\right)}{1 + \exp(\sum_{i=0}^{K} \beta_i X_i)} = 0$$

- Solve this above equation by iterative way and get the value of β_i (i=0,1,2,…,K)
- Again compute 2nd order derivatives of $l(\beta)$ that means Hessian matrix and put the estimated values of β_i. If the determinant of the Hessian matrix is less than zero, then we get the maximum value of likelihood function.

Odds Ratio: The second important statistic to study the significance is Odds Ratio. This is much easier to see which covariates (X) are actively participating in predicting the child or neonatal mortality (Y). This can be modeled unlike β_i, but using an exponent of β_i. It can be shown using the small derivation as follows: Let us assume a response variable with “j” categories and a binary

predictor (covariates) variables X that denotes whether a predictor variable is present (X_i=1) or absent (X_i=0). The odds ratio is defined as:

$$\mathrm{OR} = \frac{P_{j1}}{1-P_{j1}} \Big/ \frac{P_{j0}}{1-P_{j0}}$$

where, P_{j1} and P_{j0} denote the probabilities of the response categorical variable (binary variable of alive or death). The predictor variables may be more than two categorical. As for example, predictor variable (X) has two categorical (present vs. absent, respectively. Then the $\log_e$ odds are

$$\log_e\left(\frac{P_{j1}}{1-P_{j1}}\right) = \beta_0 + \sum_{i=1}^{K}\beta_i \quad \text{when } X_i = 1, \text{indicating predictor (covariate) is present}$$

$$\log_e\left(\frac{P_{j0}}{1-P_{j0}}\right) = \beta_0 \qquad \text{when } X_i = 0, \text{indicating predictor (covariate) is absent.}$$

Therefore, the $\log_e$ of the odds ratio can be written as

$$\log_e(\mathrm{OR}) = \log_e\left(\frac{P_{j1}}{1-P_{j1}}\right) - \log_e\left(\frac{P_{j0}}{1-P_{j0}}\right)$$

$$= \beta_0 + \sum_{i=1}^{K}\beta_i - \beta_0$$

$$= \sum_{i=1}^{K}\beta_i$$

$$= \mathrm{B}\ (\text{say})$$

Hence, $\mathrm{OR} = \exp(\mathrm{B})$. If $\mathrm{B} = 0$, then OR =1 that means the corresponding predictor variables (X) have no effect on response variable. From these we can easily calculate 95% CI for OR are given

by $\exp(B \pm SE(B))$, where SE denotes the standard error of B. In our study neonatal death and child death is treated as the response variable. If OR > 1 (with p-value < 0.05: significant), that covariate X is at high risk and vice-versa.

Appendix C: Plots and Background Characteristics of Table 1 (Input BDHS Data)

- *Serial #1 (Region)*: In Table 1, in 2011, the neonatal mortality was the highest in Sylhet (*i.e.*, 21.73%), and child mortality was the highest in Chittagong region (25.00%). On the contrary, in 2014, the neonatal and child mortality both were the highest in Sylhet region only (*i.e.*, 22.10% and 23.20%), respectively. It is worth noticing that both neonatal and child mortalities were the lowest in Khulna region (*e.g.*, 8.69% and 5.40%, respectively) in 2011 and in Barisal region (e.g., 7.90% and 7.50%, respectively) in 2014.

- *Serial #2 (Type of place)*: It is noticed that in 2011 and 2014, both neonatal and child mortalities are the highest in rural area (neo11: 69.56% and child11: 73.20%) vs. (neo14: 70.80% and child14: 72.10%). On the contrary, urban areas showed the lowest neonatal and child mortalities in both 2011 and 2014: (neo11: 30.43% and child11: 26.80%, respectively) vs. (neo14: 29.20% and child14: 27.90%, respectively).

- *Serial #3* (*Gender of child*): As far as gender of child is concerned, it is seen that both in 2011 and 2014, neonatal period, male neonatal mortality was higher compared to female neonatal mortality (male-neo11: 56.90% and female-neo11: 43.10%) vs. (male-neo14: 55.80% vs. female-neo14: 53.00%). The child mortality level (male-child11: 46.00% vs. female-child11: 54.00%) in 2011 vs. in 2014: (male-child14: 53.00% vs. female-child14: 47.00%).

- *Serial #4 (Mother's education)*: In 2011, a secondary education attribute causes neonatal mortality and child mortality to be: neo11: 41.10% and child11: 35.70%, whereas, mortality rate dropped with higher education, *i.e.*, neo11: 3.95% and child11: 1.30%. In 2014, both mortalities were the highest (neo14: 46.70% and child14: 44.20%) for secondary level of mother's education, while, with very higher mother's education, the mortality rates also dropped: neo14: 5.00% and child14: 4.70% deaths.

- *Serial #5 (Father's education)*: In 2011, father's education also indicated the similar result as mother's education, *i.e.*, with no father's education, neonatal and child mortality were higher (neo11: 31.00% and child11: 39.30%), whereas, both mortalities were lower (neo11: 8.10% and child11: 2.20%) with higher education. On the other hand, in 2014, neonatal death was higher with father's primary education (neo14: 32.90%) and child mortality was higher with father's no education (child14: 33.20%). Whereas, both mortalities were lower with higher father's education (neo14: 6.70% and child14: 6.90%).

- *Serial #6 (Mother's occupation)*: We observed that in 2011 and 2014, both neonatal and child mortalities (neo11: 92.70% and child11: 89.70%) vs. (neo14: 87.90% and child14: 87.80%) were higher for no women's occupation compared to working women's occupation (upper row) (neo11: 7.30% and child11: 10.30%) vs. (neo14: 12.10% and child14: 12.20%).

- *Serial #7 (Father's occupation)*: Father's occupation is also an important covariate for neonatal and child mortality. In 2011, both neonatal and child mortalities were the highest among farmer (neo11: 43.70% and child11: 45.10%) compared to father's occupation who were in service (neo11: 9.30% and child11: 3.60%) (Table 1). In 2014, neonatal and child mortalities were also the highest among business (neo14: 51.20% and child14: 49.20%) compared to father's occupation who were in service (neo14: 3.30% and child14: 3.40%).

- *Serial #8 (Radio)*: In 2011 and 2014, neonatal and child mortalities were the highest in families who have no radio (neo11: 93.50% and child11: 92.40%) vs. (neo14: 92.50 and child14:

92.50%), while lower mortality rate with the family who had radio (neo11: 6.50% and child11: 7.60%) vs. (neo14: 7.50% and child14: 7.50%).

- *Serial #9 (TV)*: In 2011 and 2014, both neonatal and child mortalities were the highest in families who had no TV (neo11: 69.60% and child11: 75.40%) vs. (neo14: 62.90% and child14: 63.00%) compared to the families who had TV (neo11: 30.40 % and child11: 24.60%) vs. (neo14: 37.10% and child14: 37.00%).

- *Serial #10 (Religion)*: Religion is also very important factor in relation to the mortality particularly Islamic believes. Most of the people in Bangladesh are Muslims. In 2011 and 2014, both neonatal and child mortalities were the highest in families following Muslim religion (neo11: 90.70% and child11: 89.70%) vs. (neo14: 95.00% and child14: 92.80%). On the other hand, the neonatal and child mortalities were lower in both 2011 and 2014: (neo11: 9.30% and child11: 10.30%, respectively) vs. (neo14: 5.00% and child14: 7.20%, respectively) for non-Muslims.

- *Serial #11 (Wealth index)*: It reveals that in 2011 and 2014, neonatal and child mortalities were higher for poor family (neo11: 47.00% and child11: 60.30%) vs. (neo14: 62.50% and child14: 64.90%). While in 2011, neonatal and child mortality levels were lower for middle class family (neo11: 19.00% and child11: 15.20%) vs. in 2014, both mortalities were the lowest for rich family (neo14: 18.30% and child14: 17.50%).

- *Serial #12 (SBI)*: In Table 1 shows that in 2011 and 2014, neonatal and child mortalities were found higher for less than 24 months of SBI (neo11: 87.40% and child11: 81.20%) vs. (neo14: 75.40% and child14: 72.10%). For SBI 49 and above months (neo11: 0.80% and child11: 0.40%) vs. (neo14: 7.50% and child14: 9.10%).

- *Serial #13 (Birth order)*: We observed that in 2011 and 2014, neonatal mortality was the highest (neo11: 87.40% vs. neo14: 45.80%) for first birth order. The child mortality was the highest (child11: 76.30% vs. child14: 55.80%) for 2-6 orders, while both types of mortality

rates dropped (neo11: 0.80% and child11: 4.50%) vs. (neo14: 2.50% and child14: 2.20%) for 7 and above order.

- *Serial #14 (Type of toilet facility)*: In 2011and 2014, the households that had hygienic latrines had considerably lower neonatal and child mortality (neo11: 24.50% and child11: 24.10%) vs. (neo14: 23.30% and child14: 21.60%), while, both types of mortality rates were high where the households had unhygienic latrine (neo11: 75.49% and child11: 75.89%) vs. (neo14: 76.70% and child14: 78.40%).

- *Serial #15 (Diarrhea)*: The neonatal and child mortalities in 2011 were neo11: 98.20% and child11: 97.30% for children having diarrhea disease vs. 1.80% and 2.70% for no diarrhea disease. On the other hand, in 2014, neo14: 95.00% and child14: 96.86% occur for children having diarrhea vs. 5.00% and 3.14% for no diarrhea disease.

- *Serial #16 (Mother's age)*: In 2011 and 2014, both mortalities are higher for mothers who were 15-24 years of age (neo11: 62.30% and child11: 53.60%) vs. (neo14: 53.80% and child14: 50.80%). On the contrary, for the lower mortality for mothers who were 45 and above years of age (neo11: 0.80% and child11: 0.44%) vs. (neo14: 0.80% and child14: 0.90%).

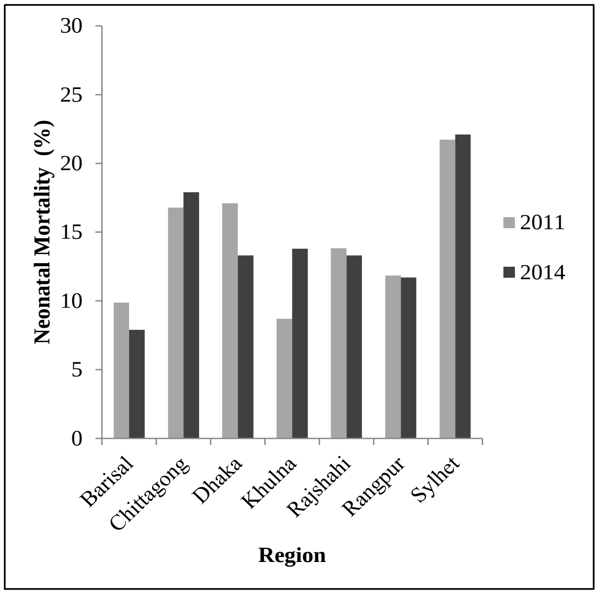

Figure 1 (a): Effect of region on neonatal mortality between 2011 and 2014.

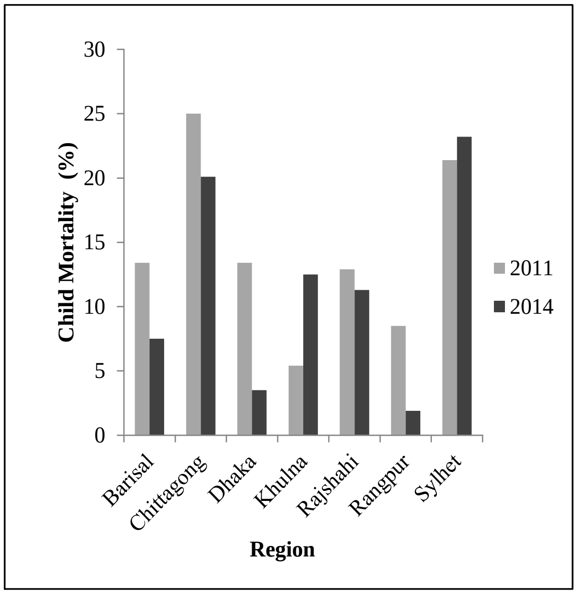

Figure 1 (b): Effect of region on child mortality between 2011 and 2014.

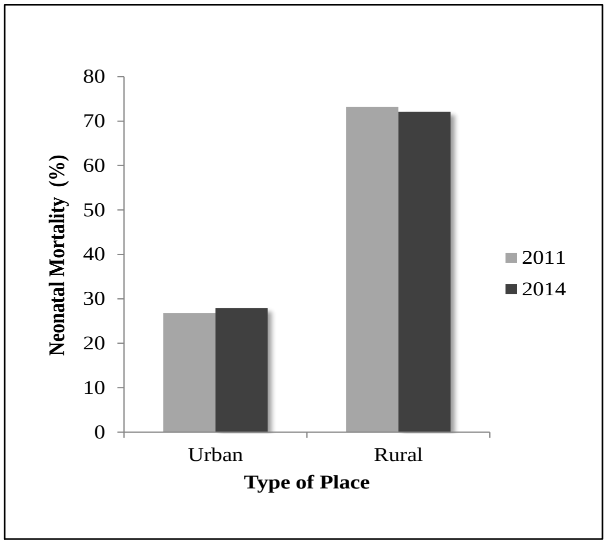

Figure 2 (a): Effect of type of place on neonatal mortality between 2011 and 2014.

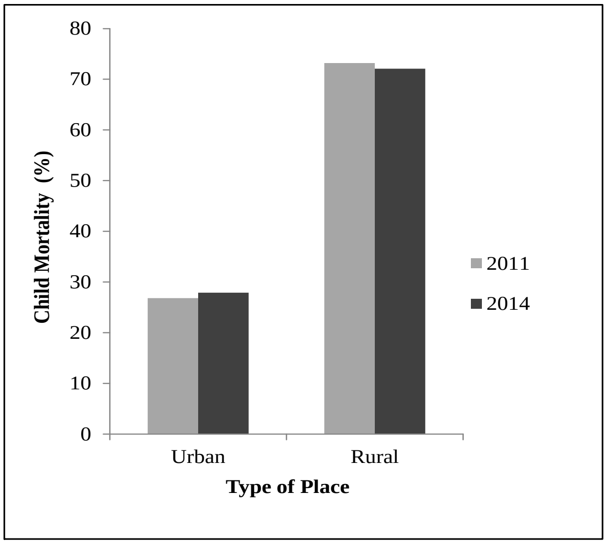

Figure 2 (b): Effect of type of place on child mortality between 2011 and 2014.

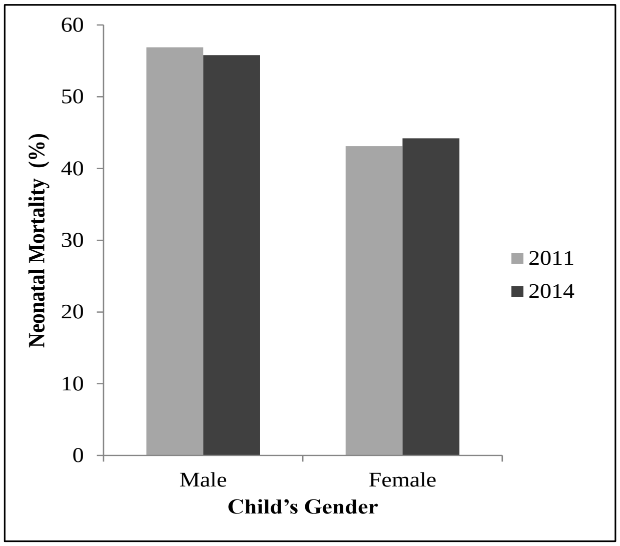

Figure 3 (a): Effect of child's gender on neonatal mortality between 2011 and 2014.

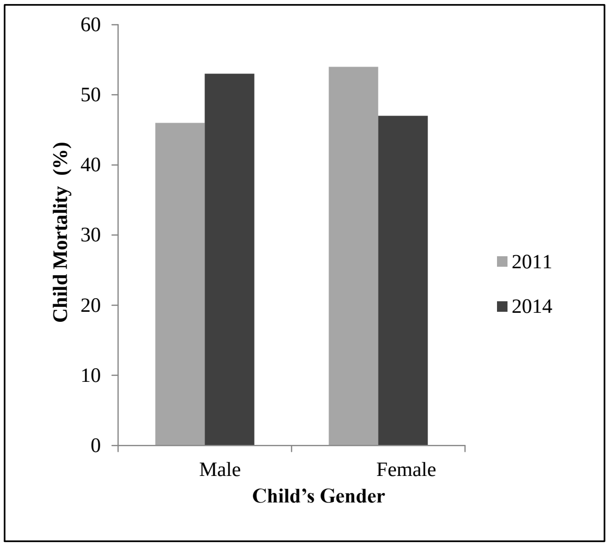

Figure 3 (b): Effect of child's gender on child mortality between 2011 and 2014.

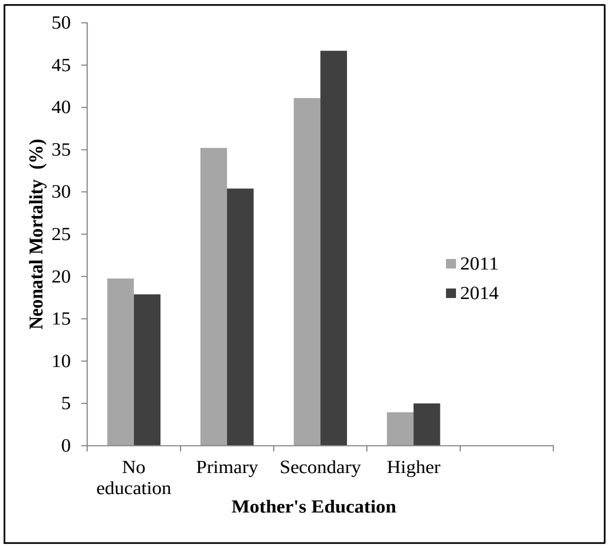

Figure 4 (a): Effect of mother's education on neonatal mortality between 2011 and 2014.

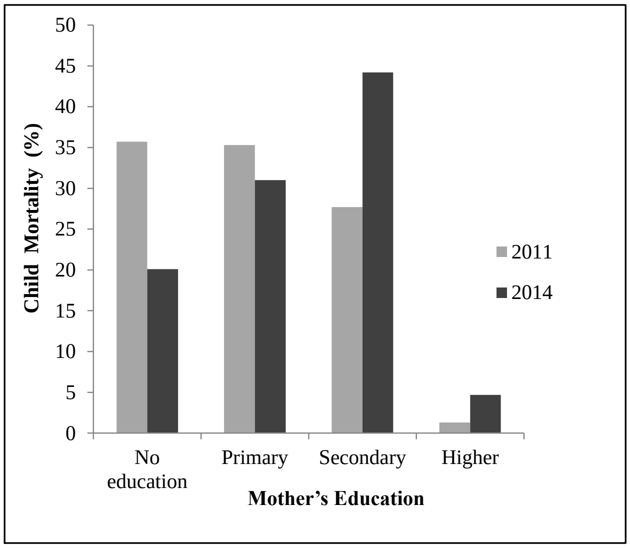

Figure 4 (b): Effect of mother's education on child mortality between 2011 and 2014.

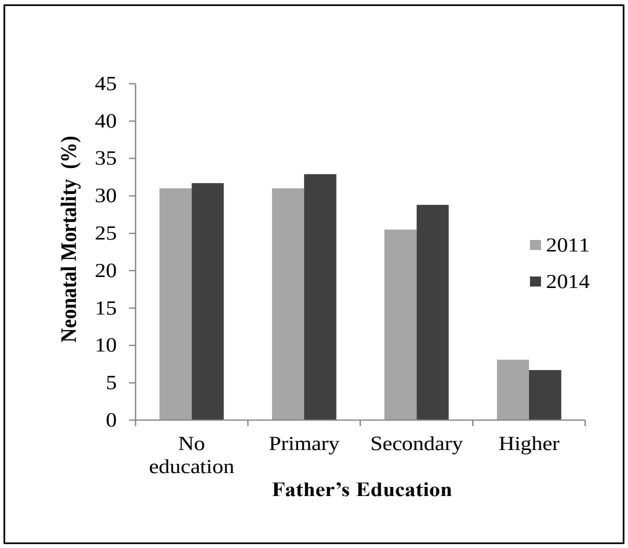

Figure 5 (a): Effect of father's education on neonatal mortality between 2011 and 2014.

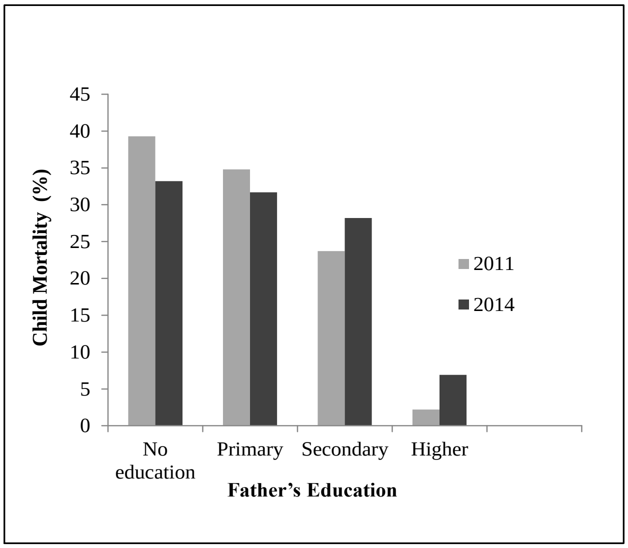

Figure 5 (b): Effect of father's education on child mortality between 2011 and 2014.

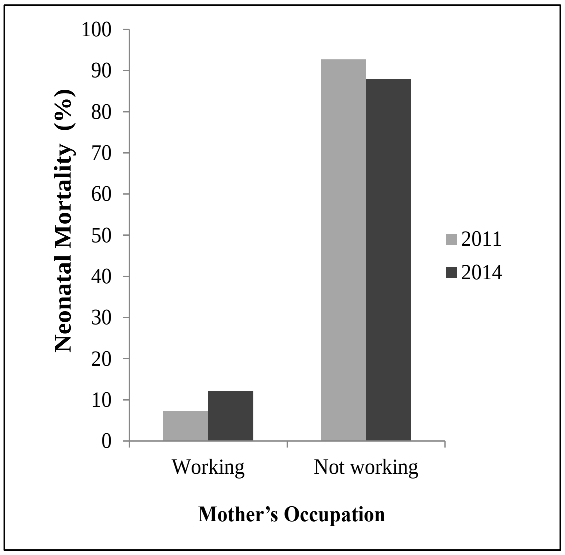

Figure 6 (a): Effect of mother's occupation on neonatal mortality between 2011 and 2014.

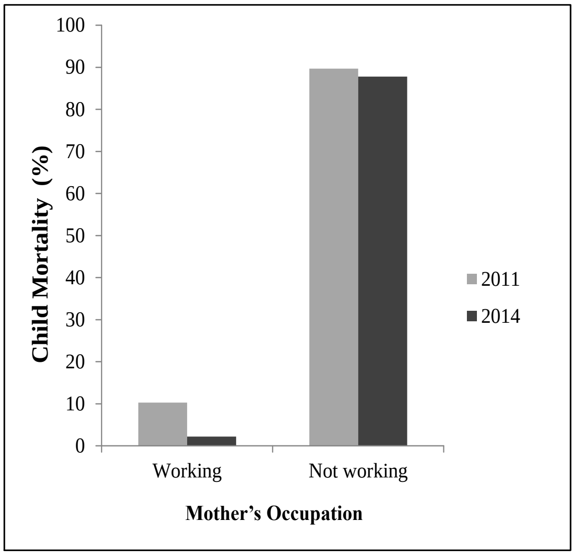

Figure 6 (b): Effect of mother's occupation on child mortality between 2011 and 2014.

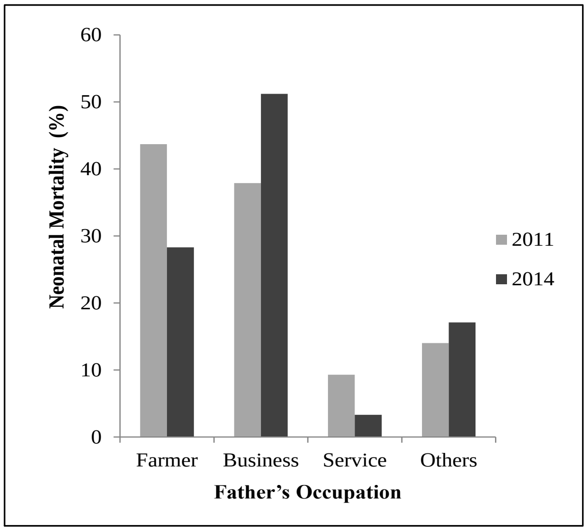

Figure 7 (a): Effect of father's occupation on neonatal mortality between 2011 and 2014.

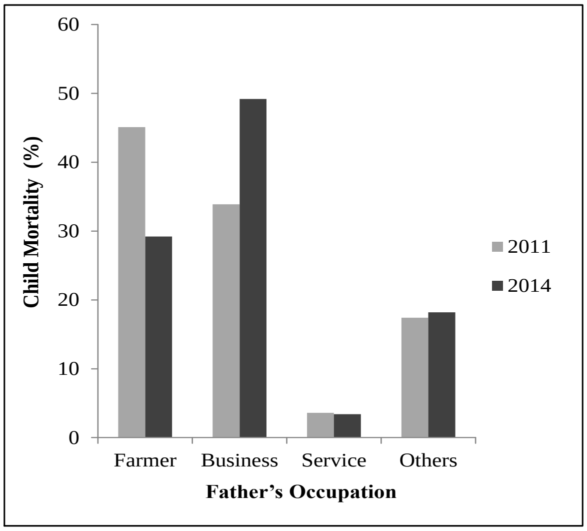

Figure 7 (b): Effect of father's occupation on child mortality between 2011 and 2014.

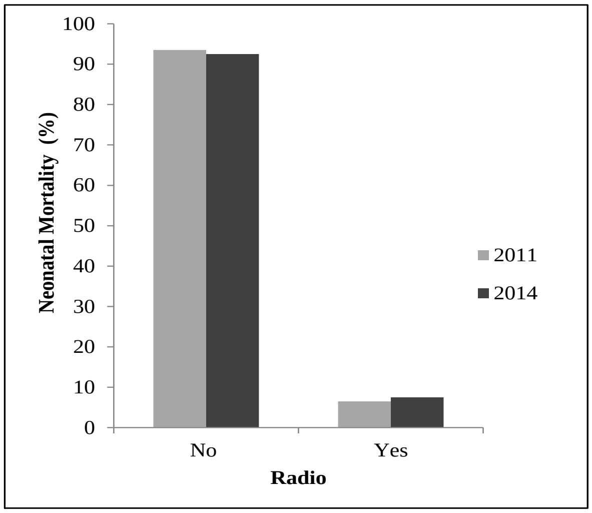

Figure 8 (a): Effect of radio on neonatal mortality between 2011 and 2014.

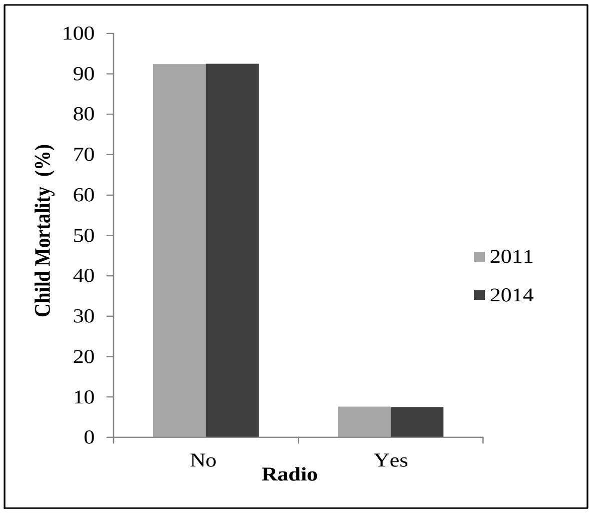

Figure 8 (b): Effect of radio on child mortality between 2011 and 2014.

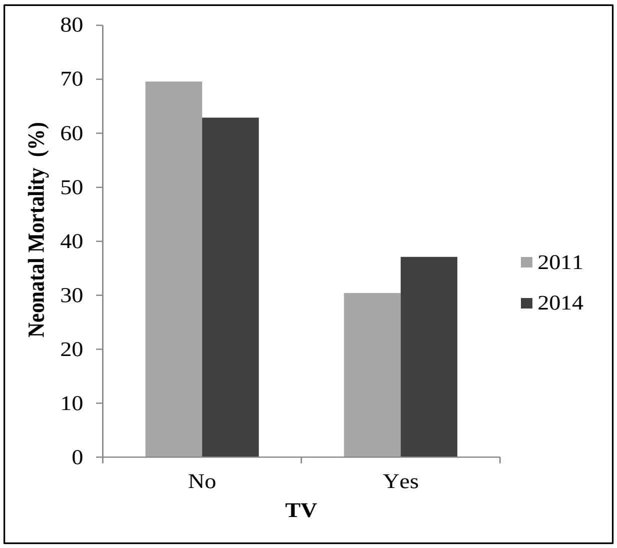

Figure 9 (a): Effect of TV on neonatal mortality between 2011 and 2014.

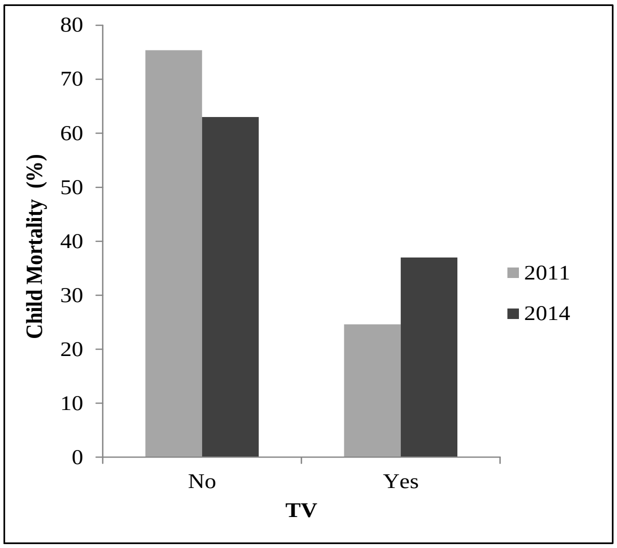

Figure 9 (b): Effect of TV on child mortality between 2011 and 2014.

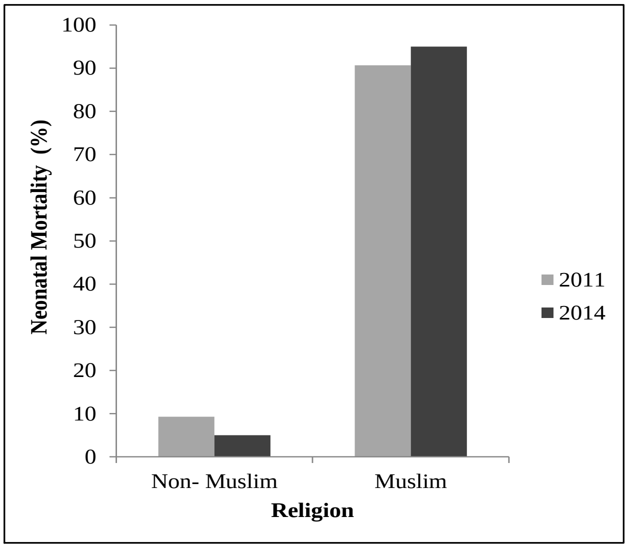

Figure 10 (a): Effect of religion on neonatal mortality between 2011 and 2014.

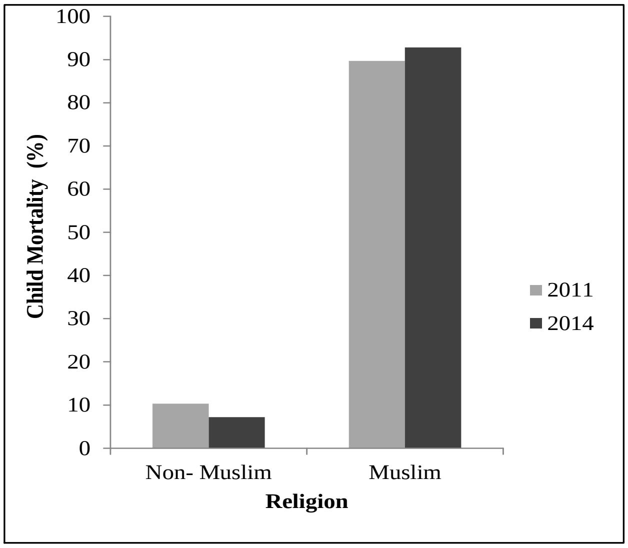

Figure 10 (b): Effect of religion on child mortality between 2011 and 2014.

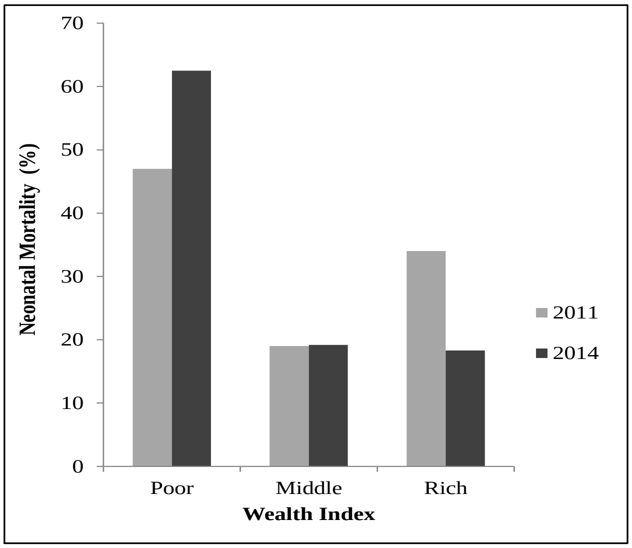

Figure 11 (a): Effect of wealth index on neonatal mortality between 2011 and 2014.

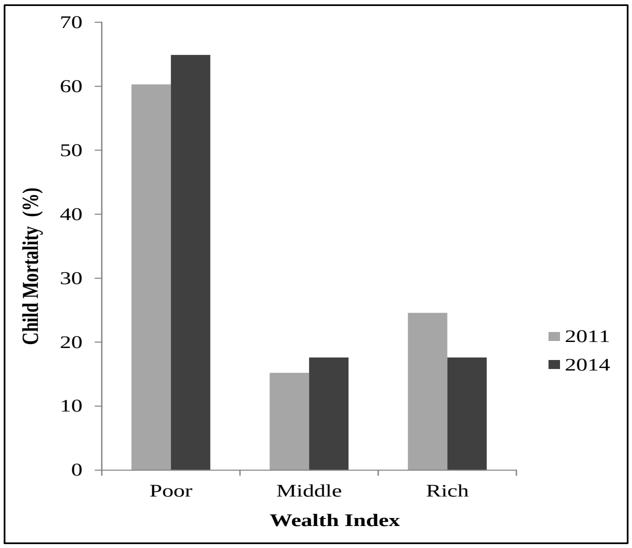

Figure 11 (b): Effect of wealth index on child mortality between 2011 and 2014.

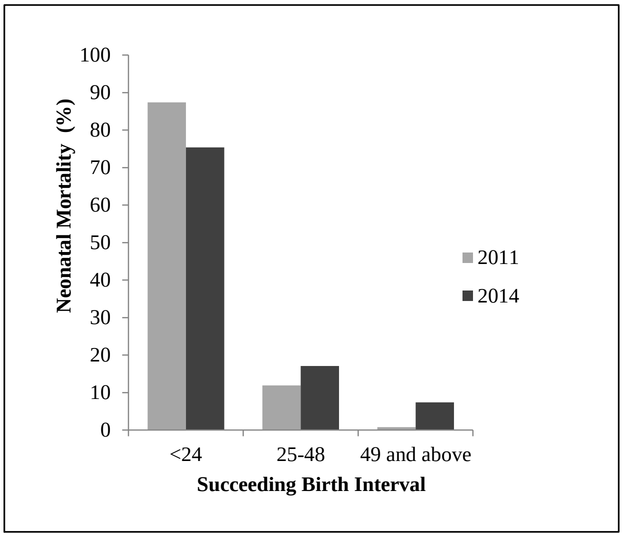

Figure 12 (a): Effect of succeeding birth interval on neonatal mortality between 2011 and 2014.

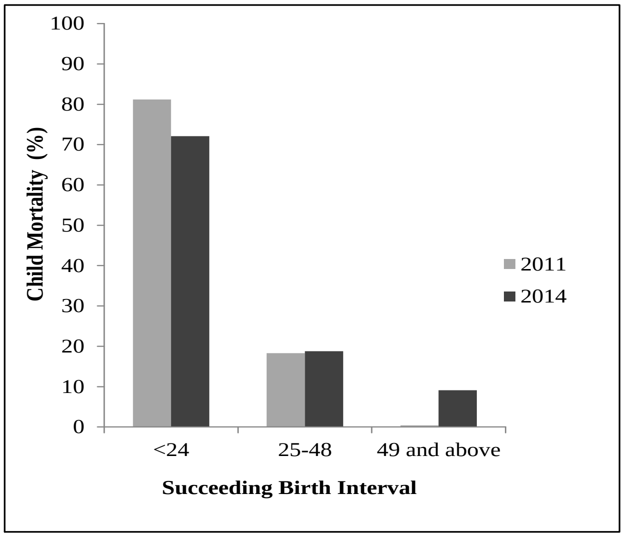

Figure 12 (b): Effect of succeeding birth interval on child mortality between 2011 and 2014.

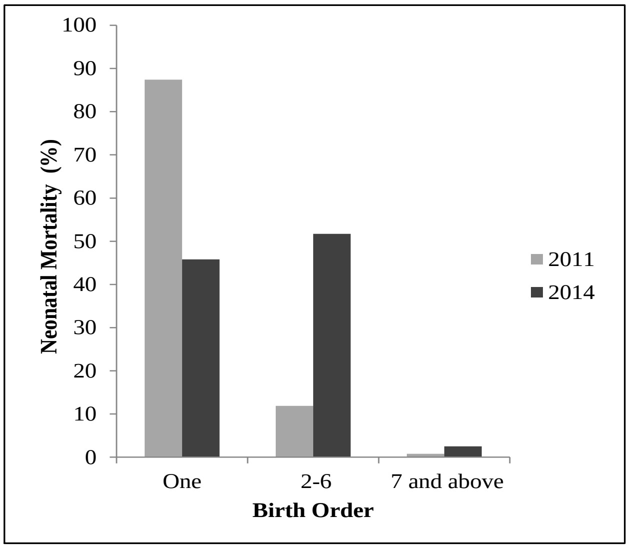

Figure 13 (a): Effect of birth order on neonatal mortality between 2011 and 2014.

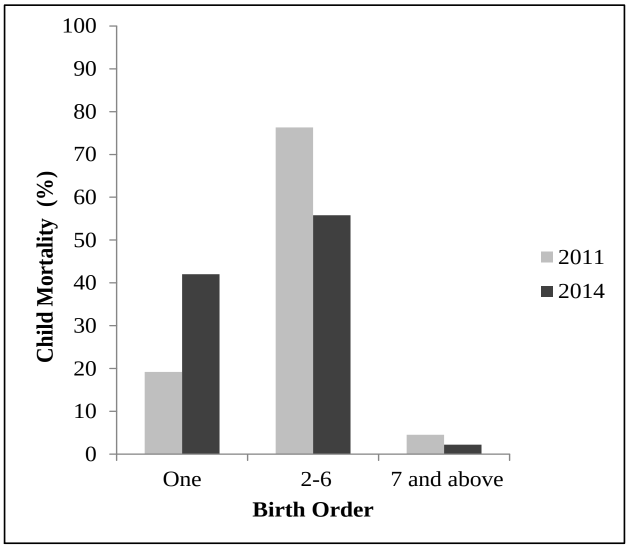

Figure 13 (b): Effect of birth order on child mortality between 2011 and 2014.

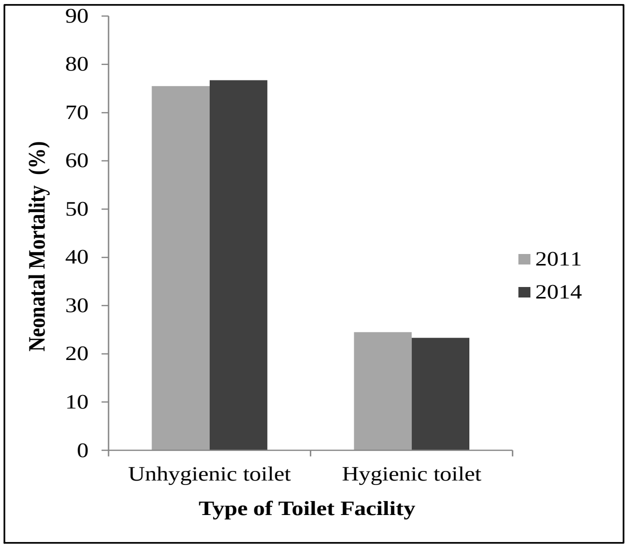

Figure 14 (a): Effect of type of toilet facility on neonatal mortality between 2011 and 2014.

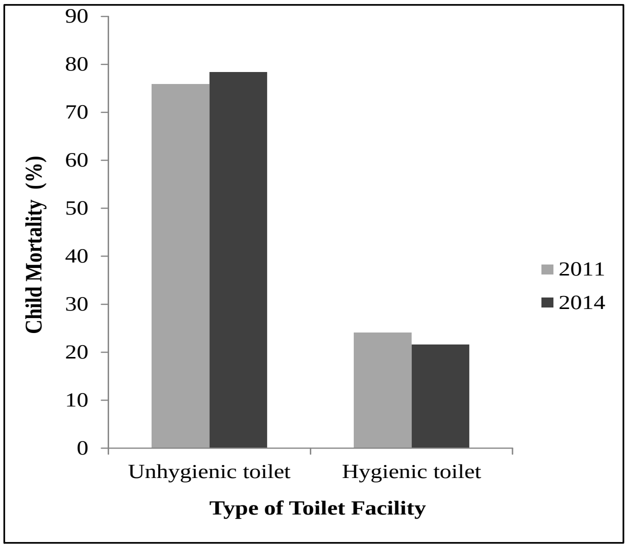

Figure 14 (b): Effect of type of toilet facility on child mortality between 2011 and 2014.

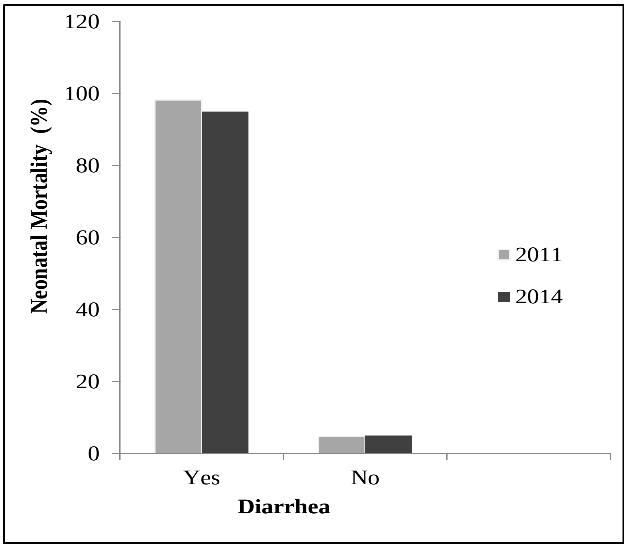

Figure 15 (a): Effect of diarrhea on neonatal mortality between 2011 and 2014.

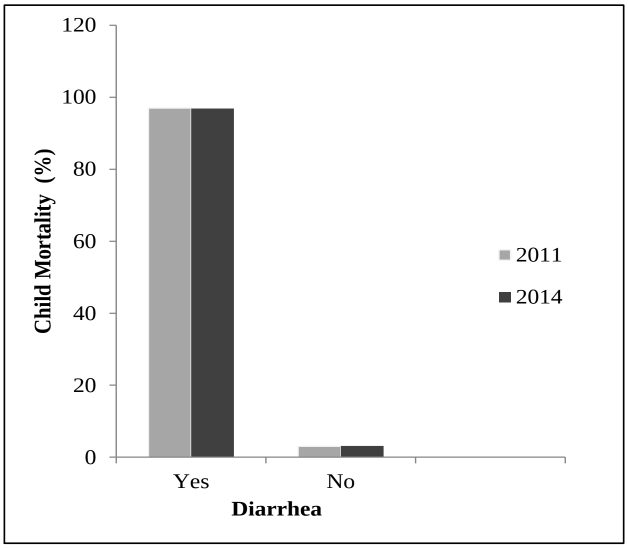

Figure 15 (b): Effect of diarrhea on child mortality between 2011 and 2014.

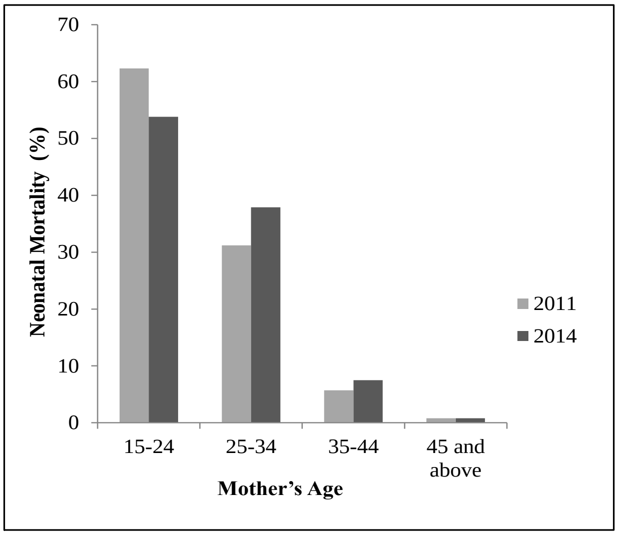

Figure 16 (a): Effect of mother's age on neonatal mortality between 2011 and 2014.

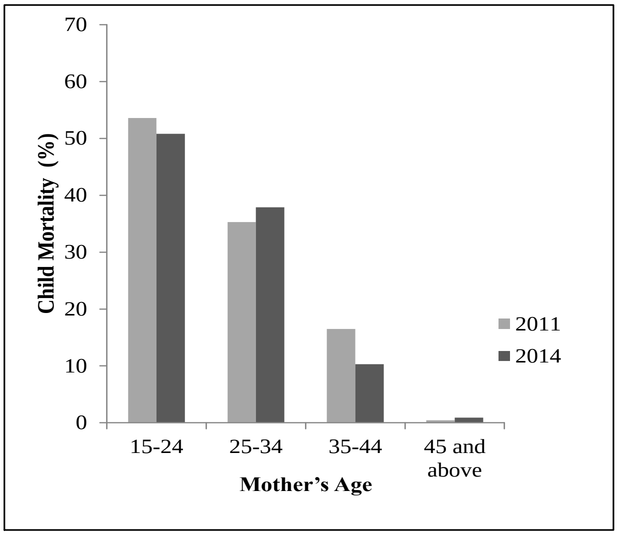

Figure 16 (b): Effect of mother's age on child mortality between 2011 and 2014.

Appendix D: Observations in Table 3 (Multiple Logistic regression estimates for the effect of the selected covariates on neonatal mortality in 2011 and 2014).

- *Serial #1 (Region)*: The finding indicates that region is significant at 1% and 5% level. In 2011, Sylhet region was 1.517 times (OR=1.517, 95% CI=1.060-2.171) higher risk of the neonatal death than Barisal region. In 2014, Sylhet, Rajshahi and Rangpur were higher risk of neonatal death than Barisal region. Rajshahi was 2.238 times (OR=2.238, 95% CI=1.098-4.563), Rangpur 2.829 times (OR=2.829, 95% CI=1.342-5.966) and Sylhet region 1.914 times (OR=1.914, 95% CI=0.996-3.679) higher risk of the neonatal death than Barisal region.

- *Serial #3 (Gender of child)*: Gender of child had significant effect on neonatal mortality. In 2011, the regression coefficient for female group was -0.275. It is clear that gender of child has a negative effect on neonatal mortality. The risk of neonatal mortality for female was 0.759 times (OR=0.759, 95% CI=0.632-0.968) lower than male. In 2014, gender of child had no effect on neonatal mortality.

- *Serial #4 (Mother's education)*: Mother's education has also significant effect on neonatal mortality. The regression coefficient for a higher educated mother is -0.054. We may observe that education status of mother has negative effect on neonatal mortality in 2011. The odds ratio of higher educated mother is 1.056 (OR=1.056, 95% CI= 0.575-1.937) that indicates 1.056 times higher risk of neonatal mortality compared to no educated mother. Here, p-value of the mother's education is greater than 1%, 5% and 10%. For that's why, mother's education has no impact on neonatal mortality in 2014.

- *Serial #5 (Father's education)*: Father's education is significant at 1% and 5% level of significance in both 2011 and 2014. In 2011, the regression coefficient for educated fathers who having secondary and higher was: -0.350 and -0.642. There is no doubt that there was a reverse relationship between father's education and neonatal mortality. Increasing education of father, neonatal mortality must be reduced and vice-versa. Fathers who had completed

secondary level and higher level they have 0.705 times (OR=0.705, 95% CI=0.532-0.935) and 0.526 (OR=0.526, 95% CI=0.324-0.853) times lower risk of the neonatal mortality compared to have no educated fathers. On the contrary, in 2014, the regression coefficient of higher educated fathers was also negative (-0.910). The odds ratio (OR) of higher educated father is 0.403. That means fathers who have higher educated; they have 0.403 (OR=0.403, 95% CI=0.163-0.992) times lower risk of neonatal mortality than no educated fathers.

- *Serial* #7 *(Father's occupation)*: The regression coefficient of father's occupation (service) was 0.281. We can say that father's occupation has positive effect on neonatal mortality. The risk of neonatal mortality for service group had 1.324 (OR=1.324, 95% CI=0.758-2.314) times higher risk compared to farmer in 2011. Whereas, in 2014, father's occupation had no effects on neonatal mortality.

- *Serial* #10 *(Religion)*: Religion was not considered a significant factor for neonatal mortality in 2011. On the other hand, since the p-value of religion is less than 0.05, so religion was a significant factor. The regression coefficient of religion was 0.070. That means religion had a positive effect on neonatal mortality in 2014. The risk of neonatal mortality in Muslim family had 0.440 times (OR=0.440, 95% CI=0.218-0.889) lower compared to non-Muslim family.

- *Serial #12 (SBI)*: In this study, it is obvious that SBI is significant at 10% level. The regression coefficient of SBI (25-48) was 0.353. So SBI had a positive effect on neonatal mortality in 2011. The odd ratio for 25-48 was 1.423, *i.e.*, 1.423 times (OR=1.423, 95% CI=1.064-1.903) higher risk compared to under 24 months of age. On the other way, in 2014, SBI had no significant effect on neonatal death.

- *Serial #13 (Birth order)*: Birth order is highly significant at 1% level of significance. The regression coefficients of birth order for 2-6 and 7 and above were 0.744 and 0.954. So there is positive impact of birth order on neonatal mortality. The odd ratio of birth order for (2-6) and 7 and above were 2.105 and 2.597, which implies that 2.105 times (OR=2.105, 95% CI=1.677-2.642) and 2.597 times (OR=2.597, 95% CI=1.300-5.186) higher risk of the neonatal

mortality compared to one of the birth order (2011). On the other way, in 2014, SBI had no significant effect on neonatal death.

- *Serial #14 (Type of toilet facility)*: It was assumed that type of toilet facility used by the children had a highly association with mother and child health behavior. Regression coefficient for families who had hygienic latrine was 0.240. It was clear that toilet facility had positive effect on neonatal mortality. In 2011, the study results (Table 3) indicated that the respondents who having hygienic toilet 1.271 times (OR=1.271, 95% CI=1.027-1.574) of higher risk of neonatal mortality than those families who had no hygienic toilet. In 2014, it was not significant.

- *Serial #15 (Diarrhea)*: Diarrhea is also a significant at 10% in 2011 and 2014. In 2011, regression coefficient for the child who had diarrhea disease was 1.130. It is clear that the child who has diarrhea disease has positive effect on neonatal mortality. The odd ratio was 0.323 for the child who had diarrhea disease. That means the neonatal death was 0.323 times (OR=0.323, 95% CI=0.165-0.632) lower risk than the child which had no diarrhea disease. In 2014, our study gives the same result as 2011.

- Serial #16 (Mother's age): In 2011, we observed that mother's age group was also a negative effect on neonatal mortality. The regression coefficients of mother's age group for 25-34 and 35-44 were -0.889 and -1.047, respectively. The odds ratios were 0.407 and 0.351 for mother's age group in 25-34 and 35-44 respectively. Thus the neonatal death occur 0.407 times (OR=0.407, 95% CI=0.326-0.508) for 25-34, age group (OR=0.351, 95% CI=0.227-0.542) times lower risk than mother age group less than 24 years. In 2014, we also observed that mother's age group was also a positive effect on neonatal mortality. The regression coefficients of mother's age group for 25-34 and 35-44 were 0.036 and 0.015, respectively. Our study showed that neonatal occurred 0.407 times for 25-34 and 0.351 times lower risk than mother age group less than 24 years.

Appendix E: Observations in Table 4 (Multiple Logistic regression estimates for the effect of the selected covariates on child mortality in 2011 and 2014).

The results of MLR for child mortality indicates that region, mother's and father's education, father's occupation, SBI, birth order and mother's age are significant at 1%, 5% and 10% level of significance. Rests of the covariates are not significant based on their p-values. The Table 4 has the following features:

- *Serial #1 (Region)*: In 2011, our finding indicates that Khulna region was significant at 5% level of significance. The regression coefficient of Khulna region was -0.774. That means Khulna region had a negative effect on child mortality. Khulna region was 0.475 times (OR=0.475, 95% CI=0.239-0.943) lower risk of the child death than Barisal region. On the other hand, Rangpur was statistically significant 1% level of significance. The regression coefficient of Rangpur region was -0.819. Rangpur region has also negative impact on child mortality. The odds ratio of Rangpur region was 0.441, which means, 0.441 (OR=0.441, 95% CI=0.243-0.802) times lower risk of child death compared to Barisal region. In 2014, the study result indicates that Khulna region was significant at 1% level of significance. Khulna region was 0.342 times lowers risk of child death compared to Barisal region. It also indicated that Rangpur, Sylhet, and Rajshahi, are significant at 1 and 5% level of significance. We observed that the regression coefficients of Khulna, Rajshahi, Rangpur, and Sylhet regions were -1.074, -0.758, -1.342, and -0.872, respectively. We also observed that Rangpur region was 0.261 times, Sylhet 0.418 times and Rajshahi 0.469 times lower risk of child death compared to Barisal region.

- *Serial #4 (Mother's education)*: In 2011, our results showed that mother's education had a negative effect on child mortality. The regression coefficients for mothers who had completed primary and secondary education were -0.454 and -0.697. The odds ratio for mother education with primary and secondary were 0.635, 0.498. That means 0.635 (OR=0.635, 95% CI=0.448-2.900) and 0.498 (OR=0.498, 95% CI=0.325-0.762) times lower risk of child mortality than

no educated mother. In 2014, it also showed that mother education had no effect on child mortality.

- *Serial #5 (Father's education)*: In 2011, regression coefficient of higher educated father was -0.355. The risk of child mortality for higher levels was 0.187 times (OR=0.187, 95% CI=0.059-0.595) lower than for no educated father. In 2014, it was also showed that a higher educated father had negative effect on child mortality. Fathers who had higher level was 2.160 times (OR=2.160, 95% CI=0.950-4.914) higher risk of child death than no educated father.

- *Serial #7 (Father's occupation)*: In 2011, father occupation had no significant effect on child mortality. On the contrary, in 2014, father's occupation had positive effect on child mortality. Here, the regression coefficients of father's occupation were 0.533 and 0.462. The risk of child mortality for business group was 1.704 times (OR=1.704, 95% CI=1.122-2.588) and others group 1.587 times (OR=1.587, 95% CI=0.963-2.614) higher compared to farmer.

- *Serial #12 (SBI)*: The regression coefficient of SBI was 0.804. That means there was positive relationship between SBI and child mortality. The odd ratio of SBI was 2.235 for 25-48 *i.e.*, 2.235 times (OR=2.235, 95% CI=1.555-3.213) higher risk than the under 24 months of age (2011). It also showed that SBI had no effect on child mortality (2014).

- *Serial #13 (Birth order)*: Birth order had positive effect on child mortality. The regression coefficients of birth order were 1.042 for 2-6 orders and 1.285 for 7 and above order. The odd ratio of birth order (2-6) was 2.835 (OR=2.835, 95% CI=1.941-4.140) times and 3.614 (OR=3.614, 95% CI=1.563-8.356) times for 7 and above higher risk of the child mortality compared to birth order one. It was also showed that birth order was not significant effect on child mortality (2014).

- *Serial #16 (Mother's age)*: In 2011, mother's age had reversed an effect on child mortality. The regression coefficients of mother's age of 25-34 and 35-44 groups were -0.769 and -0586. The odd ratio was 0.463 and 0.557 for mother's age group in 25-34, 35-44. Thus, the child

death occur 0.463 times (OR=0.463, 95% CI=0.336-0.639) for 25-34 years of age, 0.557 times (OR=0.557, 95% CI=0.331-0.937) for 35-44 years of age. In 2014, mother's age had also reversed an effect on child mortality. The regression coefficient of mother's age of 25-34 groups was -0.757. The child death occur 0.469 times (OR=0.469, 95% CI=0.233-0.943) for 25-34 years of age lower risk compared to 15-24 years of mother's age.

Appendix F: Plots of Most Significant Factors using Chi Square for Neonatal and Child Mortality: 2011 vs. 2014

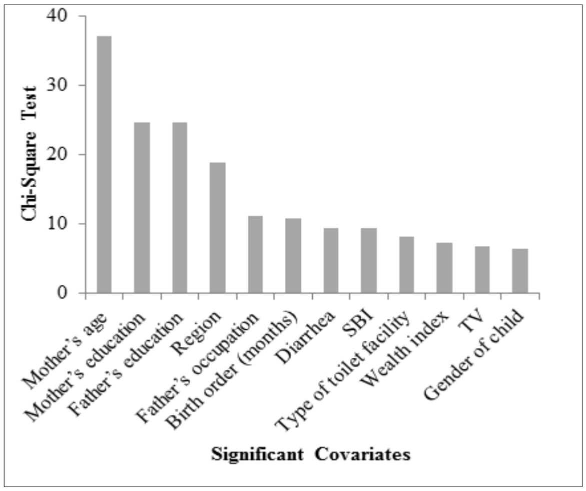

Figure 17 (a): Significant covariates for neonatal mortality in 2011.

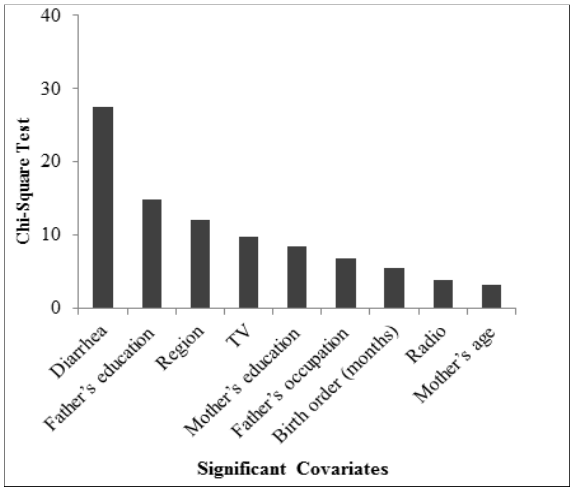

Figure 17 (b): Significant covariates for neonatal mortality in 2014.

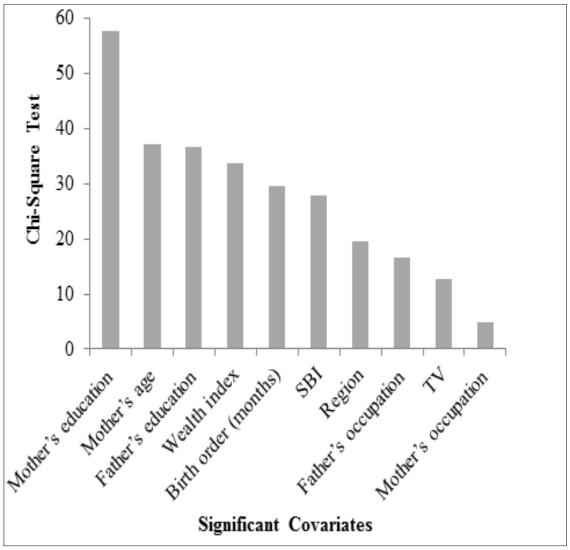

Figure 18 (a): Significant covariates for child mortality in 2011.

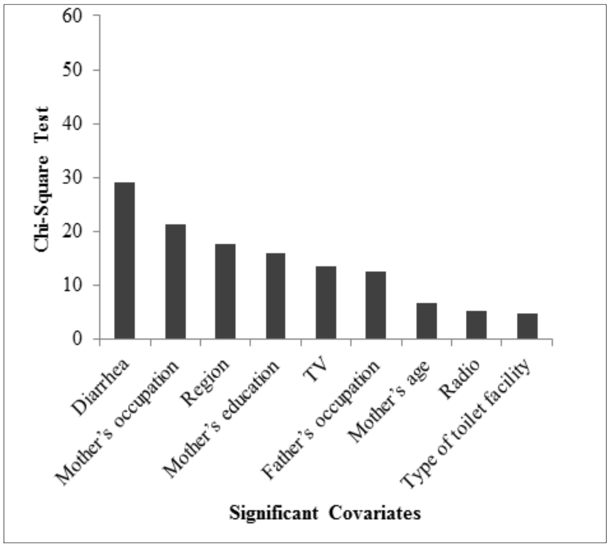

Figure 18 (b): Significant covariates for child mortality in 2014.

Appendix G: Plots of most significant factors using MLR for Neonatal and Child Mortality: 2011 vs. 2014

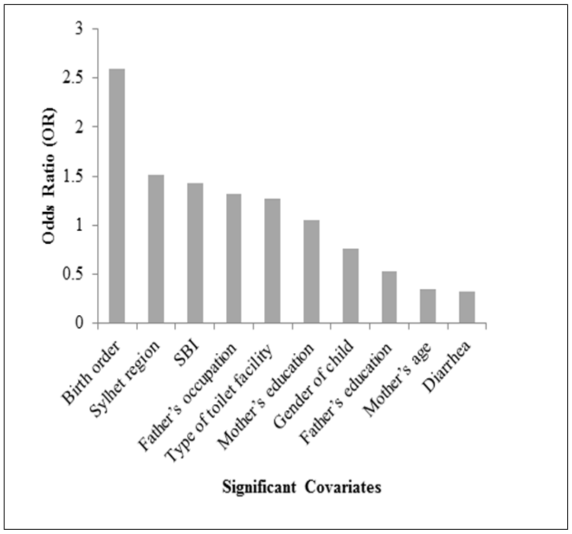

Figure 19 (a): Significant covariates for neonatal mortality in 2011.

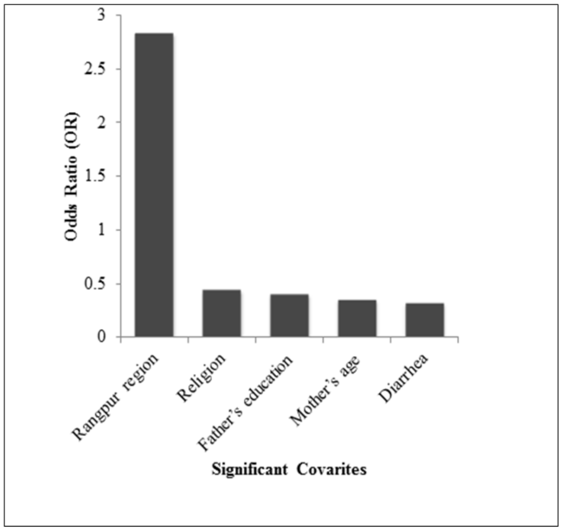

Figure 19 (b): Significant covariates for neonatal mortality in 2014.

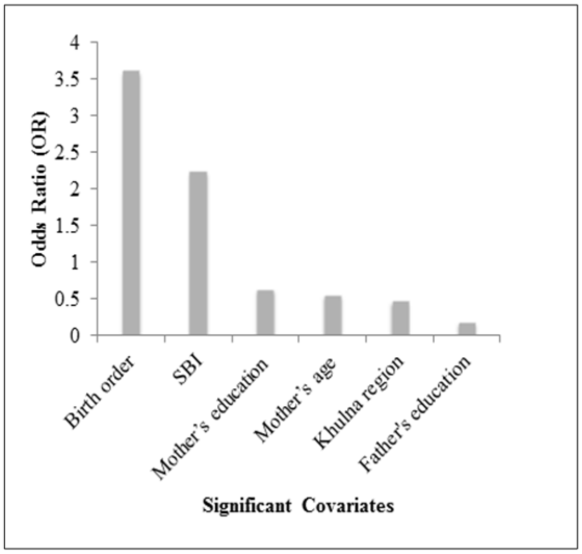

Figure 20 (a): Significant covariates for child mortality in 2011.

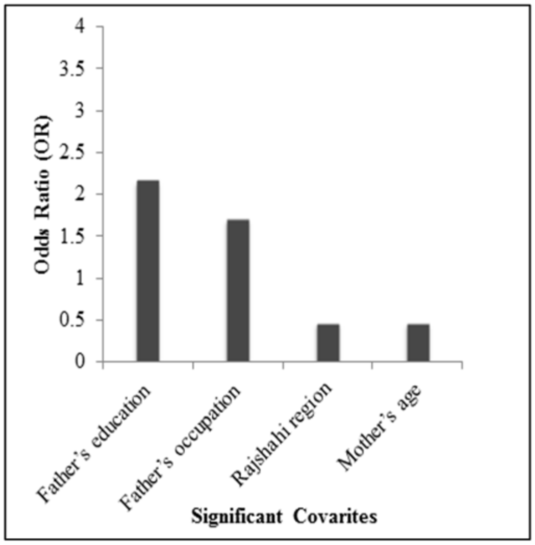

Figure 20 (b): Significant covariates for child mortality in 2014.
